# Randomised placebo-controlled trials of individualised homeopathic treatment: systematic review and meta-analysis

**DOI:** 10.1186/2046-4053-3-142

**Published:** 2014-12-06

**Authors:** Robert T Mathie, Suzanne M Lloyd, Lynn A Legg, Jürgen Clausen, Sian Moss, Jonathan RT Davidson, Ian Ford

**Affiliations:** British Homeopathic Association, Luton, UK; Robertson Centre for Biostatistics, Institute of Health and Wellbeing, University of Glasgow, Glasgow, UK; Department of Biomedical Engineering, University of Strathclyde, Glasgow, UK; Karl und Veronica Carstens-Stiftung, Essen, Germany; Homeopathy Research Institute, London, UK; Department of Psychiatry and Behavioral Sciences, Duke University Medical Center, Durham, NC USA

**Keywords:** Individualised homeopathy, Meta-analysis, Randomised controlled trials, Systematic review

## Abstract

**Background:**

A rigorous and focused systematic review and meta-analysis of randomised controlled trials (RCTs) of individualised homeopathic treatment has not previously been undertaken. We tested the hypothesis that the outcome of an individualised homeopathic treatment approach using homeopathic medicines is distinguishable from that of placebos.

**Methods:**

The review’s methods, including literature search strategy, data extraction, assessment of risk of bias and statistical analysis, were strictly protocol-based. Judgment in seven assessment domains enabled a trial’s risk of bias to be designated as low, unclear or high. A trial was judged to comprise ‘reliable evidence’ if its risk of bias was low or was unclear in one specified domain. ‘Effect size’ was reported as odds ratio (OR), with arithmetic transformation for continuous data carried out as required; OR > 1 signified an effect favouring homeopathy.

**Results:**

Thirty-two eligible RCTs studied 24 different medical conditions in total. Twelve trials were classed ‘uncertain risk of bias’, three of which displayed relatively minor uncertainty and were designated reliable evidence; 20 trials were classed ‘high risk of bias’. Twenty-two trials had extractable data and were subjected to meta-analysis; OR = 1.53 (95% confidence interval (CI) 1.22 to 1.91). For the three trials with reliable evidence, sensitivity analysis revealed OR = 1.98 (95% CI 1.16 to 3.38).

**Conclusions:**

Medicines prescribed in individualised homeopathy may have small, specific treatment effects. Findings are consistent with sub-group data available in a previous ‘global’ systematic review. The low or unclear overall quality of the evidence prompts caution in interpreting the findings. New high-quality RCT research is necessary to enable more decisive interpretation.

**Electronic supplementary material:**

The online version of this article (doi:10.1186/2046-4053-3-142) contains supplementary material, which is available to authorized users.

## Background

The nature of the research evidence in homeopathy is a matter of ongoing scientific debate. Homeopathy’s advocates tend to deny the worth of randomised controlled trials (RCTs) [1] or over-interpret their findings, whilst its critics dispute the therapy’s scientific rationale and the existence of any positive findings in the research literature [2]. There is a need to temper these divergent opinions by considering the existing RCT evidence from an objective, rigorous and transparent assessment of the research, reflecting its particular nature and intrinsic methodological quality.

Five systematic reviews have examined the RCT research literature on homeopathy as a whole, including the broad spectrum of medical conditions that have been researched and by all forms of homeopathy: four of these ‘global’ systematic reviews reached the conclusion that, with important caveats [3], the homeopathic intervention probably differs from placebo [4–7]. By contrast, the most recent global systematic review, by Shang et al., concluded there was “weak evidence for a specific effect of homeopathic remedies…compatible with the notion that the clinical effects of homeopathy are placebo effects” [8].

Four of the above reviews have distinguished RCTs of individualised homeopathy, either by mere identification [4, 8] or in formal sub-group analysis [6, 7]. In their overarching approaches, however, each of these five reviews has assessed together the RCT findings of all forms of homeopathy (individualised homeopathy, clinical homeopathy, complex homeopathy, isopathy) as if they are the same intervention. There are important differences between these therapeutic approaches, especially that individualised homeopathy typically involves a long interview between the practitioner and the patient, whereas the other three forms (non-individualised homeopathy) do not. For a placebo-controlled trial of individualised homeopathy, conclusions about ‘efficacy’ (specific effects) apply potentially to each or just some of the homeopathic medicines prescribed to the individual participants in that trial. A meta-analysis of such RCTs (including those with crossover design, which we excluded—see ‘Methods’) was published in 1998 [9], using methods that predated the current rigorous standards for conducting risk-of-bias assessments and sensitivity analysis: it reported a significant overall treatment effect that marginally was not sustained for the best-quality trials.

We aimed to clarify the results and inferences from RCTs of individualised homeopathy by conducting an up-to-date systematic review and meta-analysis to test the hypothesis: In the context of an RCT, and for the broad spectrum of medical conditions that have been researched, the main outcome of an individualised homeopathic treatment approach using homeopathic medicines is distinguishable from that of the same approach using placebos (i.e. individually prescribed homeopathic medicines have specific effects).

## Methods

Methods comply with the *PRISMA* 2009 Checklist (see Additional file 1) and with our previously published protocol [10].

### Search strategy, data sources and trial eligibility

A detailed description of the search methods used in this study has previously been published [11]. We conducted a systematic literature search to identify RCTs that compared individualised homeopathy with placebos, and for any medical condition. Each of the following electronic databases was searched from its inception up to the end of 2011, with a supplementary search of the same databases up to the end of 2013: AMED, CAM-Quest®, CINAHL, Cochrane Central Register of Controlled Trials, Embase, Hom-Inform, LILACS, PubMed, Science Citation Index and Scopus. For the 2012/13 update, CORE-Hom® was searched in addition, using the term ‘randomised’, ‘quasi-randomised’ or ‘unknown’ in the ‘Sequence generation’ field.

The full electronic search strategy for PubMed (Cochrane Highly Sensitive Search Strategy) is given in our previous paper [11]: ‘((homeopath* OR homoeopath*) AND ((randomized controlled trial [pt]) OR (controlled clinical trial [pt]) OR (randomized [tiab]) OR (placebo [tiab]) OR (clinical trials as topic [mesh:noexp]) OR (randomly [tiab]) OR (trial [ti]))) NOT (animals [mh] NOT humans [mh])’.

As stated in our published protocol [10], we then excluded trials of crossover design, of radionically prepared homeopathic medicines, of homeopathic prophylaxis, of homeopathy combined with other (complementary or conventional) intervention, or for other specified reasons. The final explicit exclusion criterion was that the trial’s team members—specifically the homeopathic practitioner/s—were not blinded to the assigned intervention. All remaining trials were eligible for systematic review.

### Outcome definitions

We identified one ‘main outcome measure’ from each study using a refinement of the approaches adopted by Linde et al. and by Shang et al. [6, 8]. Our selection of each trial’s ‘main outcome measure’ at the study end-point (i.e. at the end of scheduled follow-up, unless otherwise indicated) was based on a pre-specified hierarchical list in order of greatest to least importance, recommended by the World Health Organization (WHO) [12]. The WHO approach is an internationally accepted method to ensure that a selected outcome is the most vital to the functioning and health of the patient: it thus ensured our consistent selection of the most important and objective outcome per trial.

### Data extraction

Two reviewers (RTM and either JC, JRTD, LAL or SM) independently identified the ‘main outcome measure’ and extracted data for each trial using a standard recording approach [10]. Discrepancies in the identification and interpretation of data were discussed and resolved by consensus.

### Assessment of risk of bias

We used the domains of assessment as per the Cochrane risk-of-bias appraisal tool [13]. The extracted information enabled appraisal of freedom from risk of bias: ‘Yes’ (low risk), ‘Unclear’ (uncertain risk) or ‘No’ (high risk). We applied this approach to each of seven domains: sequence generation (domain I), allocation concealment used to implement the random sequence (II), blinding of participants and study personnel (IIIa), blinding of outcome assessors (IIIb), incomplete outcome data (IV), selective outcome reporting (V), and other sources of bias (VI). The source of any research sponsorship (i.e. potential for vested interest) was taken into account for sub-group analysis (see below), but not in risk-of-bias assessment *per se*.

Reflecting appropriately the designated ‘main outcome measure’, we rated risk of bias for each trial across all seven domains and using the following classification [10]:

Rating A = Low risk of bias in all seven domains.

Rating B*x* = Uncertain risk of bias in *x* domains; low risk of bias in all other domains.

Rating C*y.x* = High risk of bias in *y* domains; uncertain risk of bias in *x* domains; low risk of bias in all other domains.

#### Designating an RCT as ‘reliable evidence’

An ‘A’-rated trial comprises *reliable evidence*. We designated a ‘B1’-rated trial *reliable evidence* if the uncertainty in its risk of bias was for one of domains IV, V or VI only (i.e. it was required to be judged free of bias for each of domains I, II, IIIa and IIIb).

### Statistical analysis

#### Data

Mean, standard deviation and number of subjects were extracted for each continuous ‘main outcome’ and the standardised mean difference (SMD) calculated, reflecting whether a higher or a lower score was in the direction of the hypothesis favouring homeopathy; the number of favourable events and number of subjects were extracted for each dichotomous ‘main outcome’ and the odds ratio (OR) calculated. We did not adjust values to compensate for any inter-group differences at baseline.

For key meta-analyses, a single measure of ‘effect size’ was required to enable pooling of all relevant trials, and so SMD was transformed to OR using a recognised approximation method [10]. Random-effects meta-analysis models were used from the outset due to the known clinical heterogeneity among studies.

#### Study selection for meta-analysis

All RCTs that were included in the systematic review were potentially eligible for meta-analysis. If the original RCT paper did not provide or inform adequate information on our selected ‘main outcome’ to enable calculation of the SMD or the OR, we excluded the trial from meta-analysis and described the outcome as ‘not estimable’.

#### Heterogeneity and publication bias

The *I*^2^ statistic was used to assess the variability between studies; this statistic can take values between 0% and 100%, with high values indicative of strong heterogeneity.

Funnel plots and Egger’s test were used to assess publication bias [14, 15].

#### Sensitivity and sub-group analysis

Sensitivity and sub-group analyses were carried out, consistent with our published protocol [10]. The sensitivity analysis examined the impact on the pooled OR of trials’ risk-of-bias ratings. Included in sub-group analyses, we aimed to examine whether a trial’s homeopathic medicines had potency ≥12C or <12C (12-time serial dilution of 1:100 starting solution), a concentration sometimes regarded as equivalent to the ‘Avogadro limit’ for molecular dose [16].

## Results

### Included studies

The *PRISMA* flowchart from the original comprehensive literature search has been published previously [11] and comprises 489 records. The corresponding flowchart for RCT records published in 2012/13 is given in Additional file 2, which features 44 new records. A composite *PRISMA* flowchart, in standardised format, is given in Additional file 3.

Our updated literature search identified a total of five new records that were potentially eligible for the current review of RCTs that compared individualised homeopathy with placebos (Additional file 4, an update of the flowchart in our published protocol [10]). All five were single-blinded and thus ineligible for systematic review.

In total, therefore, 31 records fulfilled the relevant inclusion criteria. Data were non-extractable from 10 of those (see Additional file 4), leaving 21 records potentially available for meta-analysis. One of the included records reported findings from two RCTs. Thus, the systematic review comprises 32 RCTs, with meta-analysis of 22 of those RCTs.

### Demographic data

The 32 RCTs represented 24 different medical conditions across 12 categories (Table 1). Homeopathic potency was ≥12C in 12 trials and was not exclusively ≥12C for 20 trials (mix of >12C and <12C for 12 trials; unstated for 8 trials). Vested interest was absent in four trials, uncertain in 18 and present in 10.

**Table 1:** **Demographic data for 32 randomised controlled trials** (**RCTs**) **of individualised homeopathy**

#	First author	Year	Category	Condition	Participants’ demographics	Study setting	Potency ≥12C/ 24D	Funding source	Free of vested interest ^a^
A01	Andrade	1991	Rheumatology	Rheumatoid arthritis	Patients with active RA and fulfilling at least three pre-defined criteria	Rheumatic disease outpatient clinic, Escola Paulista de Medicina, São Paulo, Brazil	Mixed	None stated	U
A05	Bell	2004	Rheumatology	Fibromyalgia	Fibromyalgia patients	Private clinic in the USA	Mixed	External (government)	Y
A06	Bonne	2003	Mental disorder	Anxiety	Adults aged 18–65 years, of either sex, suffering from anxiety as defined by standard psychometric criteria	Department of Psychiatry, Hadassah University Hospital, Jerusalem, Israel	Y	None stated	U
A07	Brien	2011	Rheumatology	Rheumatoid arthritis	Adults formally diagnosed with RA for at least 2 years, who had relatively stable disease but some disease activity on entry	Rheumatology outpatient departments at three hospitals, UK	U	National Institute of Health Research; Samueli Institute, USA; Southampton Complementary Medicine Research Trust; Rufford Maurice Laing Foundation; Dreluso Pharmazeutika GmBH; National Health Service Fund for Science	Y
A09	Cavalcanti	2003	Dermatology	Uraemic pruritus	Dialysis for more than 6 months, moderate to severe pruritus, absence of other causes for pruritus	Haemodialysis centres, Rio de Janeiro state, Brazil	Mixed	None stated	U
A10	Chapman	1999	Neurology	Brain injury	Patients with mild traumatic brain injury	University medical school in the USA	Y	External (government; foundation; hom pharm providing all meds)	N
A11	de Lange de Klerk	1994	Respiratory infection	URTI	Children aged 1.5–10 years, with at least three upper respiratory tract infections in the past year or with two such episodes plus otitis media with effusion at the time of entry examination	Paediatric outpatient department of university hospital, Amsterdam, Netherlands	Mixed	Dutch Ministry of Welfare, Cultural Affairs, and Public Health	Y
A13	Fisher	2006	Dermatology	Eczema	Adults aged 18–65 years, of either sex, with planned treatment for dermatitis at the RLHH	Royal London Homoeopathic Hospital (RLHH), London, UK	Mixed	None stated	U
A14	Frass	2005	Surgery and anaesthesiology	Sepsis	Patients with severe sepsis	Intensive Care Unit at a university hospital in Austria	Y	None stated	U
A16	Gaucher	1994	Tropical disease	Cholera	Patients with cholera, in a state of dehydration requiring parenteral treatment	University of San Marcos, Lima, Peru	U	None stated; several acknowledged, including employee of hom pharm company	U
A18	Jacobs	1993	Gastroenterology	Childhood diarrhoea	Children aged 0.5–5 years, with history of acute diarrhoea	Participants’ homes, Leon, Nicaragua	Y	Boiron Research Foundation, Norwood, Pennsylvania, USA,	U
A19	Jacobs	1994	Gastroenterology	Childhood diarrhoea	Children with a history of acute diarrhoea	Nicaragua	Y	External, including hom pharm that might have provided the remedies for the trial	U
A20	Jacobs	2001	Ear, nose and throat	Otitis media (acute)	Patients with acute otitis media	Children’s clinic in the USA	Y	External (hom pharm—all remedies—research grant)	N
A21	Jacobs	2000	Gastroenterology	Childhood diarrhoea	Children with a history of acute diarrhoea	Nepal	Y	External (hom research foundation)	U
A22	Jacobs	2005b	Obstetrics and gynaecology	Menopause post breast cancer	Women with history of carcinoma or stage I–III breast cancer who had completed all surgery, chemotherapy and radiation treatment and who averaged at least three hot flushes per day for previous month	Private medical clinic, Seattle, USA	U	External (charity); meds donated by hom pharm	N
A23	Jacobs	2005a	Mental disorder	ADHD	Children, aged 6–12 years, meeting DSM-IV criteria for ADHD	Private homeopathic clinic in Seattle, USA	U	External (government); meds donated by hom pharm	N
A24	Jansen	1992	Gastroenterology	Proctocolitis	Active proctocolitis (proven by endoscopy and histology)	Outpatient dept, Maria Hospital, Tilburg, Netherlands	Y	None stated	U
A25	Kainz	1996	Dermatology	Warts	Children aged 6–12 years, with common warts on the back of hands	Department of Dermatology, University of Graz, Austria	Mixed	Firma Spagyra, Groding, Austria: ‘kindly provided’ homeopathic medicines and placebos. Otherwise, none stated	N
A26	Katz	2005	Mental disorder	Depression	Adults, aged 18–80 years, of either sex, suffering from major depressive episode of moderate severity as defined by DSM-IV	Lower Clapton group practice, east London, UK	U	Homeopathic Research Committee; Blackie Foundation Trust. Verum and placebo homeopathic medicines donated by Laboratoires Boiron	N
A30	Naudé	2010	Mental disorder	Insomnia	Insomnia sufferers (identified through local advertising)	South Africa	Mixed	None stated	U
A31	Rastogi **(**a**)**	1999	Immune disorder	HIV	Adults aged 18–50 years, of either sex, with positive antibody reaction to HIV-1 or HIV-2 or both confirmed by repeat ELISA and/or Western blot	Regional Research Institute for Homoeopathy, Mumbai, India	Mixed	None stated	U
A31	Rastogi **(**b**)**	1999	Immune disorder	HIV	Adults aged 18–50 years, of either sex, with positive antibody reaction to HIV-1 or HIV-2 or both confirmed by repeat ELISA and/or Western blot	Regional Research Institute for Homoeopathy, Mumbai, India	Mixed	None stated	U
A32	Sajedi	2008	Neurology	Cerebral palsy	Children aged 1–5 years, with mild to moderate spasm due to cerebral palsy	Saba Clinic (Developmental Disorder Center), Tehran, Iran	U	Internal	U
A33	Siebenwirth	2009	Dermatology	Eczema	Adults aged 18–35 years with at least a 1-year history of atopic dermatitis (>20% of skin surface)	Klinik und poliklinik für Dermatologie und Allergologie am Biederstein, Munich, Germany	Mixed	The study medications gifted by Deutsche Homöopathie-Union (DHU). Funded by Karl und Veronica Carstens-Stiftung	N
A34	Steinsbekk	2005	Respiratory infection	URTI	Children, under 10 years old, who had been to a medical doctor for URTI	In children’s own homes, recruited mainly from those previously diagnosed with URTI when attending a hospital casualty department	Y	External: Norwegian Research Council. Homeoden Belgium made the medicines	U
A35	Straumsheim	2000	Neurology	Migraine	Adults aged 18–65 years, of either sex, diagnosed according to International Headache Society classification criteria	Arena Medisinske Senter, Oslo, Norway	Mixed	DCG Farmaceutia (Gotenborg): supplied all homeopathic medicines. Norwegian Research Council	N
A36	Thompson	2005	Obstetrics and gynaecology	Menopause post breast cancer	Women who attended oncology centre for breast cancer, did not have metastatic disease, were not on any other treatment and experiencing more than three hot flushes per day	Outpatient department of an NHS homeopathic hospital, UK	Y	External (charity; hom pharm—‘provided’ all remedies, presumably via hospital pharmacy dept)	U
A37	Walach	1997	Neurology	Headache	Patients diagnosed according to the International Headache Society classification criteria	Germany	Mixed	Homeoden (Gent) and Gudjons (Augsburg): supplied homeopathic medicines	U
A38	Weatherley-Jones	2004	Mental disorder	Chronic fatigue syndrome	Adults with chronic fatigue	Two outpatient departments in UK	U	External (charity)	Y
A39	White	2003	Allergy and asthma	Childhood asthma	Children, aged 5–15 years, with diagnosis of asthma and prescription for beta agonist and/or corticosteroid inhaler issued within previous 3 months	Five general practices in market towns in Somerset, UK	U	External, including hom pharm that provided all remedies	N
A40	Whitmarsh	1997	Neurology	Migraine	Adults aged 18–60 years, of either sex, with definite diagnosis of migraine by pre-defined criteria	Princess Margaret Migraine Clinic, Charing Cross Hospital, London, UK	Y	Nelsons Ltd; Homeopathic Medical Research Council; Blackie Foundation Trust	U
A41	Yakir	2001	Obstetrics and gynaecology	Premenstrual syndrome	Women aged 20–50 years, with diagnosis of PMS according to pre-defined criteria; symptomatology corresponding to one of five homeopathic medicines	Gynaecology outpatient clinic, Hadassah University Hospital, Jerusalem, Israel	Y	Dolisos Laboratories; Deutsche Homeopathie Union; named individuals	N

The complete bibliographic details for the studies included are found in Additional file 4. *Y* yes, *U* unclear, *N* no, *RA* rheumatoid arthritis, *URTI* upper respiratory tract infection. ^a^Vested interest: support (direct, through research sponsorship; indirect, via gifted medicines) from a company that provided homeopathic medicines for the trial.

### Summary of findings

For each trial, Table 2 includes details of the sample size, the identified ‘main outcome measure’ (and whether dichotomous or continuous), the end-point and whether the study was described in the original report as a ‘pilot’ (or ‘preliminary’ or ‘feasibility’) study. The median sample size (for *N* = 32 trials) was 43.5 (inter-quartile range 27 to 67). There were 28 different ‘main outcome measures’ and for an end-point that ranged from 12 h to 12 months.

**Table 2:** **Summary of findings table**

#	First author	Year	Pilot	ITT sample	PP sample	PP sample > median(43.5)	Attrition rate(%)	‘Main’ outcome identified	Nature of ‘main’ outcome	End-point
A01	Andrade	1991	N	44	33	N	25.0	Ritchie articular index	Continuous	6 months
A05	Bell	2004	Y	62	53	Y	14.5	Tender point pain on palpation	Continuous	3 months
A06	Bonne	2003	N	44	39	N	11.4	Hamilton Rating Scale for Anxiety (HAM-A)	Continuous	10 weeks
A07	Brien	2011	N	32	23	N	28.1	Proportion of patients meeting ACR 20% improvement criteria (‘ACR20 response’)	Dichotomous	28 weeks
A09	Cavalcanti	2003	N	28	20	N	28.6	Responders: patients with more than 50% reduction in pruritus score	Dichotomous	60 days
A10	Chapman	1999	Y	61	50	Y	18.0	SRH-SLPD functional assessment tool: SRS (symptoms) sub-scale	Continuous	4 months
A11	de Lange de Klerk	1994	N	175	170	Y	2.9	Daily total symptom score	Continuous	1 year
A13	Fisher	2006	Y	38	27	N	28.9	VAS of overall symptom severity	Continuous	13 weeks
A14	Frass	2005	N	70	67	Y	4.3	Patient survival	Dichotomous	180 days
A16	Gaucher	1994	N	80	51	Y	36.3	Degree of dehydration	Continuous	12 h
A18	Jacobs	1993	N	34	33	N	2.9	Number of days until fewer than 3 unformed stools for 2 consecutive days	Continuous	Up to 6 days
A19	Jacobs	1994	N	92	81	Y	12.0	Number of days until fewer than 3 unformed stools for 2 consecutive days	Continuous	Up to 5 days
A20	Jacobs	2001	Y	75	75	Y	0.0	Treatment failure	Dichotomous	5 days (cumulative total)
A21	Jacobs	2000	N	126	116	Y	7.9	Number of days until fewer than 3 unformed stools for 2 consecutive days	Continuous	5 days
A22	Jacobs	2005b	Y	53	33	N	37.7	Hot flash severity score	Continuous	12 months
A23	Jacobs	2005a	Y	43	37	N	14.0	Conners Global Index–Parent (CGI-P)—total	Continuous	17 weeks
A24	Jansen	1992	Y	10	4	N	60.0	Endoscopic appearance (grade)	Continuous	12 months
A25	Kainz	1996	N	67	60	Y	10.4	Responders: patients with at least 50% reduction in area of skin affected by warts	Dichotomous	8 weeks
A26	Katz	2005	Y	7	3	N	57.1	Hamilton Depression Scale (HAMD)	Continuous	12 weeks
A30	Naudé	2010	N	33	30	N	9.1	Sleep Impairment Index (SII) summary score	Continuous	4 weeks
A31	Rastogi **(**a**)**	1999	N	50	42	N	16.0	CD4+ T-lymphocyte counts	Continuous	6 months
A31	Rastogi **(**b**)**	1999	N	50	38	N	24.0	CD4+ T-lymphocyte counts	Continuous	6 months
A32	Sajedi	2008	N	24	16	N	33.3	Modified Ashworth Scale: measurement of muscle tone in *right leg*	Continuous	4 months
A33	Siebenwirth	2009	N	24	14	N	41.7	MP (multiparameter dermatitis) score	Continuous	32 weeks
A34	Steinsbekk	2005	N	251	199	Y	20.7	Parent-reported URTI total symptom score	Continuous	Duration of 12 weeks
A35	Straumsheim	2000	N	73	68	Y	6.8	Frequency of migraine attacks per month	Continuous	Last month of 4-month period
A36	Thompson	2005	Y	53	45	Y	15.1	MYMOP profile score	Continuous	16 weeks
A37	Walach	1997	N	98	92	Y	6.1	Frequency of headaches per month	Continuous	Last month of 3-month period
A38	Weatherley**-**Jones	2004	N	103	86	Y	16.5	Responders: those with clinical improvement (Multidimensional Fatigue Inventory: mental fatigue)	Dichotomous	7 months
A39	White	2003	N	89	74	Y	16.9	Childhood Asthma Questionnaire (CAQ) sub-scale for severity of symptoms	Continuous	52 weeks
A40	Whitmarsh	1997	N	63	60	Y	4.8	Frequency of migraine attacks per month	Continuous	Last month of 4-month period
A41	Yakir	2001	Y	23	19	N	17.4	Menstrual distress questionnaire (MDQ) score	Continuous	Duration of 3 months (last 7 days per cycle)

*ITT* intention to treat, *PP* per protocol*, Y* yes, *N* no.

### Risk of bias and reliable evidence

Table 3 provides the risk-of-bias details for each of the 32 trials and sub-divided by (a) included in our meta-analysis and (b) not included in our meta-analysis. Domain IIIa (blinding of participants and trial personnel) contributed the least risk of bias overall, with no trial classed ‘high risk’. Domain IV (completeness of outcome data) presented the greatest methodological concerns, with 14 trials judged ‘high risk’. Domain II (allocation concealment) presented the most uncertain methodological judgments, with 21 (66%) trials assessed ‘unclear risk’ and 10 (31%) assessed ‘low risk’. A risk-of-bias bar graph is shown in Additional file 5.

**Table 3:** **Risk**-**of**-**bias assessments for trials**: (**a**) **included in meta**-**analysis** and (**b**) **not included in meta**-**analysis**

#	First author	Year	Risk-of-bias domain							Risk of bias	Risk- of -bias rating
			I	II	IIIa	IIIb	IV	V ^a^	VI		
**(a):** **Included in meta-** **analysis**											
A11	de Lange de Klerk	1994	U	U	U	U	Y	Y	Y	Uncertain	B4
A19	Jacobs	1994	Y	Y	Y	Y	U	Y	Y	Uncertain^b^	B1
A25	Kainz	1996	U	U	U	U	U	Y	U	Uncertain	B6
A10	Chapman	1999	Y	U	Y	Y	Y	Y	Y	Uncertain	B1
A35	Straumsheim	2000	U	U	Y	Y	Y	Y	Y	Uncertain	B2
A20	Jacobs	2001	Y	Y	Y	Y	U	Y	Y	Uncertain^b^	B1
A41	Yakir	2001	U	U	Y	Y	U	Y	Y	Uncertain	B3
A06	Bonne	2003	U	U	Y	Y	Y	Y	U	Uncertain	B3
A05	Bell	2004	Y	Y	Y	Y	Y	Y	U	Uncertain^b^	B1
A14	Frass	2005	Y	U	Y	U	Y	Y	Y	Uncertain	B2
A23	Jacobs	2005a	Y	U	Y	Y	Y	Y	Y	Uncertain	B1
A36	Thompson	2005	Y	U	Y	Y	Y	Y	Y	Uncertain	B1
A40	Whitmarsh	1997	U	U	Y	U	Y	U	N	High	C1.4
A31	Rastogi (a)	1999	U	U	U	U	N	N	U	High	C2.5
A31	Rastogi (b)	1999	U	U	U	U	N	N	U	High	C2.5
A09	Cavalcanti	2003	U	U	Y	Y	N	Y	U	High	C1.3
A38	Weatherley-Jones	2004	Y	U	Y	Y	N	Y	Y	High	C1.1
A22	Jacobs	2005b	Y	Y	Y	Y	N	Y	Y	High	C1.0
A13	Fisher	2006	Y	U	Y	Y	N	Y	U	High	C1.2
A32	Sajedi	2008	U	U	U	U	N	Y	Y	High	C1.4
A33	Siebenwirth	2009	U	Y	Y	Y	N	Y	N	High	C2.1
A07	Brien	2011	Y	Y	Y	Y	N	Y	Y	High	C1.0
**(b):** **Not included in meta-analysis**											
A01	Andrade	1991	U	U	U	U	N	N	U	High	C2.5
A24	Jansen	1992	U	U	U	U	N	N	N	High	C3.4
A18	Jacobs	1993	U	U	Y	Y	U	N	U	High	C1.4
A16	Gaucher	1994	N	N	U	U	N	N	U	High	C4.3
A37	Walach	1997	U	Y	Y	U	Y	N	N	High	C2.2
A21	Jacobs	2000	Y	Y	Y	Y	Y	N	N	High	C2.0
A39	White	2003	Y	Y	Y	Y	Y	N	Y	High	C1.0
A26	Katz	2005	Y	U	Y	U	N	N	N	High	C3.2
A34	Steinsbekk	2005	Y	Y	Y	Y	N	N	Y	High	C2.0
A30	Naudé	2010	Y	U	Y	Y	U	N	Y	High	C1.2

Trials are arranged chronologically within their risk-of-bias rating category.

^a^Unless a published study protocol was available, completeness of reporting (domain V) was judged solely on correspondence of ‘Results’ with details in ‘Methods’ section of paper.

^b^Reliable evidence.

*Y* yes, *U* unclear, *N* no (regarding freedom from risk of bias).

Table 3(a): No trial was ‘A’-rated (low risk of bias overall)—i.e. none fulfilled the criteria for all seven domains of assessment. Table 3(a) therefore includes a list of 12 trials that were classed uncertain risk of bias (‘B’-rated) and 10 that were classed high risk of bias (‘C’-rated). Table 3(a) also shows the three ‘B1’-rated trials that satisfied our criteria for *reliable evidence*. All other trials had unclear or high risk of bias in important methodological aspects and may be regarded as non-reliable evidence.

Table 3(b): Trials that were deficient in domain V (selective outcome reporting) included the ten whose data were not extractable for meta-analysis and which were thus ‘C’-rated by default; seven of these ten trials were already ‘C’-rated due to deficiency in at least one other domain of assessment.

### Meta-analysis

**Figure 1:**
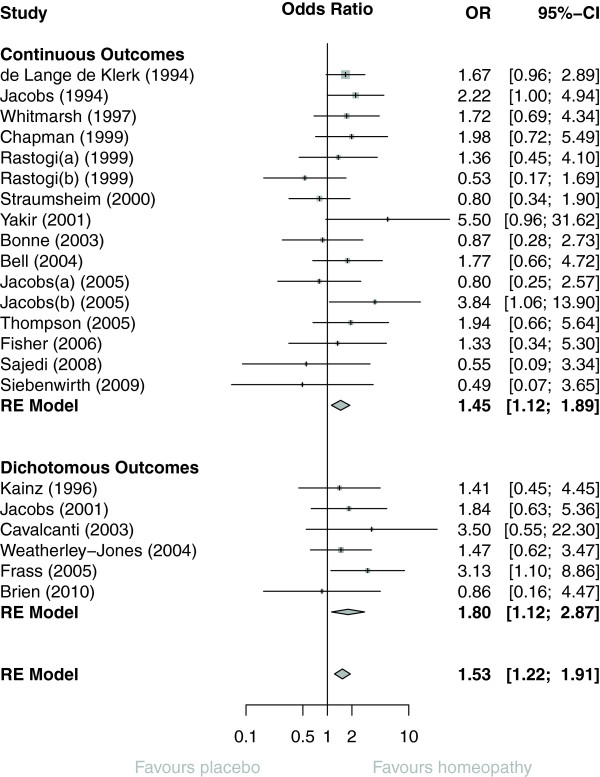
**Forest plot showing odds ratio (OR) and 95% confidence interval (CI) for each of 22 RCTs of individualised homeopathy, with pooled OR (random-effects [RE] model) for trials with continuous outcomes, dichotomous outcomes, and for all 22 RCTs.**

The data extracted per trial (continuous or dichotomous data) are tabulated in Additional file 6. Figure 1 illustrates the OR for each trial; the original SMD data for each of the studies with continuous data are shown in Additional file 7. Of the 22 trials, 15 had an effect favouring homeopathy (i.e. OR > 1), 3 of them statistically significantly; 7 trials had an effect favouring placebo, none of them significantly. Total sample size = 1,123 (*N* = 22 trials).

Pooled OR was 1.53 (95% confidence interval (CI) 1.22 to 1.91; *P* < 0.001). There was no difference depending on whether the ‘main outcome’ was continuous (OR = 1.45; 95% CI 1.12 to 1.89) or dichotomous (OR = 1.80 (95% CI 1.12 to 2.87); *P* = 0.44).

#### Heterogeneity and publication bias

**Figure 2:**
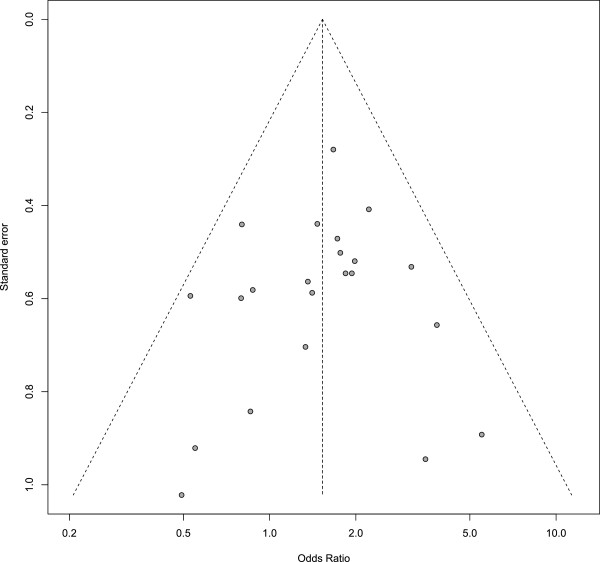
**Funnel plot: 22 RCTs of individualised homeopathy.**

Despite the clinical heterogeneity across the studies, the statistical heterogeneity between the studies was low (*I*^2^ = 0% [95% CI 0% to 40%]), and therefore the variability in the estimated pooled OR is also relatively low. No evidence of publication bias was apparent from the funnel plot (Figure 2) or from Egger’s test (*P* = 0.59).

#### Risk of bias and reliable evidence

**Figure 3:**
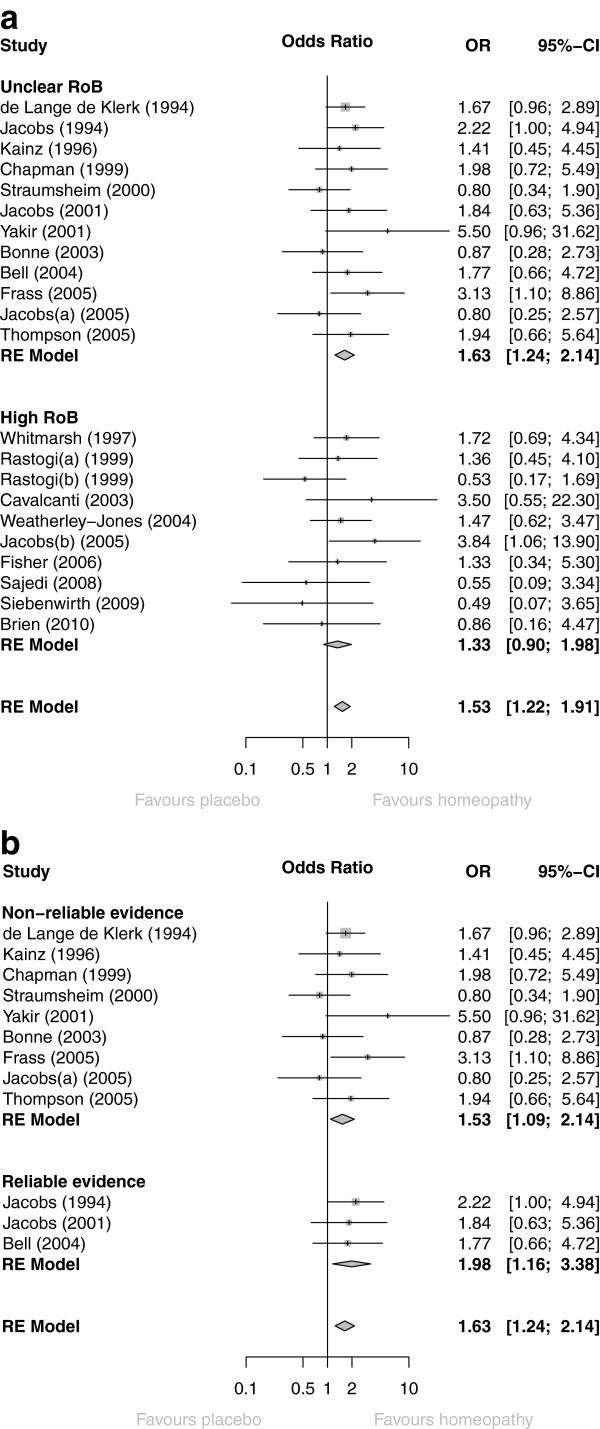
**Forest plots showing odds ratio (OR) and 95%cconfidence interval (CI) for each of (a) 22 RCTs of individualised homeopathy, with pooled OR (random-effects [RE] model) for trials with unclear risk of bias (RoB), high RoB, and for all 22 RCTs; (b) 12 ‘B’-rated RCTs of individualised homeopathy, with pooled OR (RE model) for trials with non-reliable evidence, reliable evidence, and for all 12 RCTs.**

Figure 3a shows the OR data for all 22 trials, grouped by their ‘B’- or ‘C’-rating. There was no difference between ‘B’- and ‘C’-rated trials: *P* = 0.41.

- ‘Uncertain risk of bias’ (all ‘B’-rated; *N* = 12): OR = 1.63 (95% CI 1.24 to 2.14; *P* < 0.001);
- ‘High risk of bias’ (all ‘C’-rated; *N* = 10): OR = 1.33 (95% CI 0.90 to 1.98; *P* = 0.15).

Figure 3b shows the OR data for the 12 ‘B’-rated trials, grouped by reliability of evidence. There was no difference between the two sub-sets (*P* = 0.42).

- Uncertain risk of bias/reliable evidence (*N* = 3): OR = 1.98 (95% CI 1.16 to 3.38; *P* = 0.013).
- Uncertain risk of bias/non-reliable evidence (*N* = 9): OR = 1.53 (95% CI 1.09 to 2.14; *P* = 0.014).

#### Sensitivity analysis

**Figure 4:**
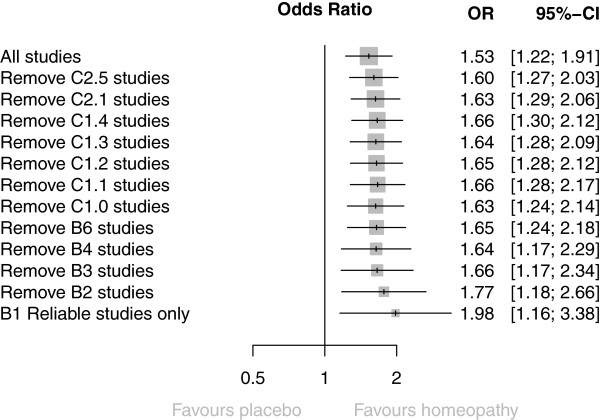
**Sensitivity analysis, showing progressive effect on pooled odds ratio (OR) of removing data by trials’ risk-of-bias rating.**

Figure 4 shows the effect of removing data by trials’ risk-of-bias rating; full details are given in Additional file 8. The set of six trials rated ‘B1’ has been sub-divided by those with/without reliable evidence. The pooled OR showed a statistically significant effect in favour of homeopathy for every value of *N*, down to and including the final *N* = 3 trials with reliable evidence. Additional file 8 also states whether a trial was included in the ‘global’ analyses by Linde and/or Shang [6, 8]: of the 22 RCTs we subjected to meta-analysis, 8 had previously been analysed and 14 had not (our selected ‘main outcome measure’ differs for 3 of the 8 trials also included by Shang—Additional file 9).

#### Sub-group analyses

**Figure 5:**
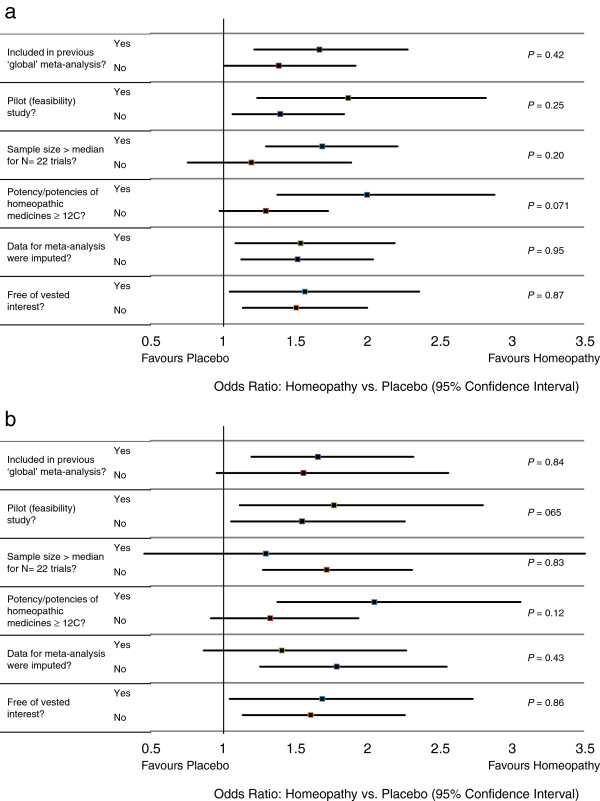
**Interactions between sub**-**groups for (a) all** ***N*** **= 22 trials with analysable data and (b)** ***N*** **= 12** **‘B’-** **rated trials.**

Pooled OR favoured homeopathy for all sub-groups and was statistically significant for all but two of them (<median; potency not exclusively ≥12C): Figure 5a. There was no evidence of difference in the pooled statistic between any sub-groups. Similar results were seen for the *N* = 12 (Figure 5b) and *N* = 3 analyses (data not illustrated). Full details are given in Additional file 10. We observed a statistically significant pooled OR, favouring homeopathy, for the eight trials that we have in common with those previously reported by Shang et al. [8].

## Discussion

Twenty-nine of the 32 trials had unclear or high risk of bias in important domains of assessment. Poor reporting or other deficiencies in the original papers prevented data extraction for meta-analysis from 10 of the 32 trials; the potential influence of the 10 on our overall meta-analysis is unknown, but because of their intrinsic low quality, their absence does not alter our principal conclusions. High and unclear risk of bias featured almost equally in our 22-trial analysis; thus, the overall quality of analysed evidence was low or unclear, necessitating caution in interpreting the findings.

As was the case for the previous ‘global’ systematic reviews of homeopathy RCTs that have included meta-analysis [6, 8], there are obvious limitations in pooling data from diverse medical conditions, outcome measures and end-points. Thus, a given pooled effects estimate here does not have a clear numerical meaning or relative clinical value: it is a summary measure that enables statistical significance and mean ‘effect size’ to be attributed and to be interpreted in testing an hypothesis that individually prescribed homeopathic medicines have specific effects.

Though our conclusions can be made most securely from three trials with reliable evidence, this sub-set of studies is too small to enable a decisive answer to our tested hypothesis. Equivocal RCT evidence of this nature is not unusual in medical science, in which conclusions are commonly based on just two eligible RCTs per systematic review [17]. Given the specific focus of our study, a statistically significant OR of 1.98 may be interpreted as a small ‘effect size’ for these three trials collectively and does not differ significantly from the ‘effect size’ observed in our analysis of 22 trials (OR = 1.53). Such ‘effect sizes’ seem comparable with, for example, sumatriptan for migraine, fluoxetine for major depressive disorder and cholinesterase inhibitors for dementia [18]. The detection of a small yet significant pooled OR, with the perspective that only a few single trials showed statistically significant effects, supports conjecture that the impact of an individualised homeopathic prescription may be difficult to observe readily in the context of any one particular placebo-controlled trial [19, 20].

Our approach to quality assessment was objective and internally consistent, and the methodological implications of our risk-of-bias findings are the same as those for RCTs in conventional medicine. Indeed, it is noteworthy that domain II (allocation concealment) had the fewest ‘low risk of bias’ assessments (for 31% of our trials): this compares historically with adequately described allocation concealment in just 16% conventional medicine trials that were published during the period 1960 to 1995 [21].

Two of the three trials with reliable evidence used medicines that were diluted beyond the Avogadro limit. Our pooled effects estimate for the three trials, therefore, is either a false positive or it reflects the relevance of new hypotheses about the biological mechanism of action of homeopathic dilutions [22, 23]. It should also be noted that one of these same three trials displayed evidence of vested interest. It remains to be seen if our assessments of model validity [24] support or refute the legitimacy of these three trials as currently the most important contributors to the evidence base in individualised homeopathy.

### Comparison with previous systematic reviews

Our database is different from that of both Linde and Shang [6, 8]. Firstly, we concentrated solely on peer-reviewed trials of individualised homeopathic treatment. Secondly, we have an updated set of trials for meta-analysis: 14 of our 22 RCTs were not included in the previous ‘global’ analyses. Thirdly, our selected ‘main outcome measure’—and thus our calculated OR—differs for three of the eight previously analysed trials of individualised homeopathy. Fourthly, our group of three RCTs with ‘reliable evidence’ is founded on a *more* exacting standard than for Shang’s ‘trials of higher methodological quality’: indeed, by Shang’s explicit criteria for domains IV, V and VI, we would label five of our ‘non-reliable’ trials [20, 25–28] as ‘higher methodological quality’.

A notable finding from sub-group analysis is that our 14 newly examined trials do not differ in ‘effect size’ from the eight that were included in previous ‘global’ meta-analysis by Shang et al. [8], disputing suggestions that the evidence base in homeopathy is weakening with time [29]. Noteworthy too is the significantly positive pooled OR that we observed for those eight trials and the close similarity of its value to that calculable from Shang’s forest plot data for the same eight [30].

Like us, Linde and colleagues reported a pooled OR of 1.50–2.00 for the highest-quality RCTs [3, 6]. In their separate analysis of individualised homeopathy, however, Linde and Melchart noted a smaller ‘effect size’ whose statistical significance was only marginally not sustained for the highest-quality trials [9]. Unlike our predecessors, we found no evidence that lower-quality trials displayed a larger treatment effect than that of higher-quality studies: indeed, our ten ‘C’-rated trials with extractable data displayed a non-significant pooled effects estimate. It may be that our stringent judgmental approach led to a less extreme range of quality assessments than those of the earlier reviewers. Importantly, we found no evidence of publication bias, removing any need for data adjustment.

## Conclusions

- There was a small, statistically significant, treatment effect of individualised homeopathic treatment that was robust to sensitivity analysis based on ‘reliable evidence’.
- Findings are consistent with sub-group data available in a previous ‘global’ systematic review of homeopathy RCTs.
- The overall quality of the evidence was low or unclear, preventing decisive conclusions.
- New RCT research of high quality on individualised homeopathy is required to enhance the totality of reliable evidence and thus enable clearer interpretation and a more informed scientific debate.

## Authors’ information

The study is intrinsic to the work of the British Homeopathic Association through its Research Development Adviser, RTM; it was assisted by a grant from the Manchester Homeopathic Clinic (see below). Trustees and staff of neither charity contributed to the design, analysis or write-up. RTM devised and led the study in collaboration with his co-authors.

## Electronic supplementary material

Additional file 1: **Checklist.** *PRISMA* 2009 Checklist. (DOC 63 KB)

Additional file 2: ***PRISMA*** **flowchart for records published in 2012 or 2013.** (DOC 100 KB)

Additional file 3: ***PRISMA*** **flowchart for all records published up to and including 2013.** (DOC 58 KB)

Additional file 4: **Details of records of RCTs of individualised homeopathy.** *Asterisk*: paper reports two RCTs. *Double asterisk*: trial reported as double-blinded, but revealed to be single-blinded on inspection of published protocol. *SD* standard deviation. Reference numbering continues from previously published listings [11]. (DOCX 60 KB)

Additional file 5: **Risk-of-bias bar-graph for 32 RCTs of individualised homeopathy.** (DOCX 15 KB)

Additional file 6: **Data extracted for meta-analysis of RCTs with: (a) continuous main outcome measure and (b) dichotomous main outcome measure.** (DOCX 19 KB)

Additional file 7: **Standardised mean difference (SMD) and 95% confidence interval (CI) for RCTs with continuous main outcome measure, showing pooled statistic (random effects [RE] model).** (PDF 6 KB)

Additional file 8: **Sensitivity analysis on risk-of-bias rating, and including specified demographic data per trial.** (DOCX 24 KB)

Additional file 9: **Meta-analysed trials in common with those included by Shang et al.** [8] **, showing comparison of quality assessment and degree of similarity of selected outcome measure.** (DOCX 14 KB)

Additional file 10: **Sub-group analysis showing interaction for: (a) all** ***N*** **= 22 trials with analysable data, (b)** ***N*** **= 12 (‘B’-rated) trials with uncertain risk of bias and (c) sub-set of (‘B1’-rated) trials with reliable evidence.** (DOCX 22 KB)

## Abbreviations

CI: Confidence interval
OR: Odds ratio
RCT: Randomised controlled trial
SMD: Standardised mean difference
WHO: World Health Organization.
