# Supplementary material for: Randomised placebo-controlled trials of individualised homeopathic treatment: systematic review and meta-analysis
Source: Syst Rev. 2014 Dec 6;3:142. doi: 10.1186/2046-4053-3-142 (PMC4326322; doi:10.1186/2046-4053-3-142)
Supplement: Supplementary file 2 — Additional file 2: PRISMA flowchart for records published in 2012 or 2013. (DOC 100 KB) [file 13643_2014_328_MOESM2_ESM.doc]

**Additional file 2**

**35 unique records identified by search in online databases**

**9 unique records identified through other sources**

***SCREENING***

**44 records**

____________

**Book chapters, theses, etc.:**

**2 non-repeats**

**(R227–228)**

**0 repeats**

***FULL TEXT***

**42 journal records**

**Abstracts, etc.:**

**9 non-repeats**

**(R229–237)**

**1 repeat**

**(R238)**

**32 substantive journal records**

**Substantive journal records: 0 repeat publications**

**32 non-repeat, substantive, journal records**

**2**

**not randomised &/or not contr.**

**(R239–240)**

**2**

**not randomised &/or not contr.**

**(R242–243)**

**5**

**non peer-reviewed,**

**non-repeat, substantive, journal records**

**27**

**peer-reviewed,**

**non-repeat, substantive, journal records**

**1**

**not ‘homeopathic’**

**(R241)**

**0**

**not ‘homeopathic’**

**8**

**OTP-controlled**

**16**

**placebo-controlled**

**1**

**placebo-controlled**

**2**

**OTP-controlled**

**5**

**indiv. hom.**

**(A264–268)**

**11**

**non-indiv. hom.**

**(A269–279)**

**0**

**indiv. hom.**

**0**

**indiv. hom.**

**5**

**indiv. hom.**

**(A280–284)**

**2**

**non-indiv. hom.**

**(A289–290)**

**1**

**non-indiv. hom.**

**(A288)**

**3**

**non-indiv. hom.**

**(A285–287)**

*Reference numbering continues from previously published listings* [10]

**References**

| A264 | Adler UC, Krüger S, Teut M, Lüdtke R, Schützler L, Martins F, Willich SN, Linde K, Witt CM (2013). Homeopathy for depression: A randomized, partially double-blind, placebo-controlled, four-armed study (DEP-HOM). PLoS ONE; 8: e74537. |
| --- | --- |
| A265 | Chakraborty PS, Lamba CD, Nayak D, John MD, Sarkar DB, Poddar A, Arya JS, Raju K, Vivekanand K, Singh HBK, Baig H, Prusty AK, Singh V, Nayak C (2013). Effect of individualized homoeopathic treatment in influenza like illness: A multicenter, single blind, randomized, placebo controlled study. Indian J Res Homoeopathy; 7: 22-30. |
| A266 | Chakraborty PS, Varanasi R, Majumdar AK, Banoth K, Prasad S, Ghosh MS, Sinha MN, Reddy GRC, Singh V, Nayak, C (2013). Effect of homoeopathic LM potencies in acute attacks of haemorrhoidal disease: A multicentric randomized single-blind placebo-controlled trial. Indian J Res Homoeopathy; 7: 72-80. |
| A267 | Oberai P, Gopinadhan S, Varanasi R, Mishra A, Singh V, Nayak C (2013). Homoeopathic management of attention deficit hyperactivity disorder: A randomised placebo-controlled pilot trial. Indian J Res Homoeopathy; 7: 158-167. |
| A268 | Saha S, Koley M, Hossain SI, Mundle M, Ghosh S, Nag G, Datta AK, Rath P (2013). Individualized homoeopathy versus placebo in essential hypertension: A double-blind randomized controlled trial. Indian J Res Homoeopathy; 7: 62-71. |
| A269 | Bell IR, Howerter A, Jackson N, Brooks AJ, Schwartz GE (2012). Multiweek resting EEG cordance change patterns from repeated olfactory activation with two constitutionally salient homeopathic remedies in healthy young adults. J Altern Complement Med; 18: 445-453. |
| A270 | Bell IR, Howerter A, Jackson N, Aickin M, Bootzin RR, Brooks AJ (2012). Nonlinear dynamical systems effects of homeopathic remedies on multiscale entropy and correlation dimension of slow wave sleep EEG in young adults with histories of coffee-induced insomnia. Homeopathy; 101: 182-192. |
| A271 | Bell IR, Brooks AJ, Howerter A, Jackson N, Schwartz GE (2013). Acute electroencephalographic effects from repeated olfactory administration of homeopathic remedies in individuals with self-reported chemical sensitivity. Altern Ther Health Med; 19: 46-57. |
| A272 | Colau JC, Vincent S, Marijnen P, Allaert FA (2012). Efficacy of a non-hormonal treatment, BRN-01, on menopausal hot flashes: a multicenter, randomized, double-blind, placebo-controlled trial. Drugs R D; 12: 107-119. |
| A273 | Dean ME, Karsandas R, Bland JM, Gooch D, MacPherson H (2012). Homeopathy for mental fatigue: lessons from a randomized, triple blind, placebo-controlled cross-over clinical trial. BMC Complement Altern Med; 12: 167. |
| A274 | Harrison CC, Solomon EM, Pellow J (2013). The effect of a homeopathic complex on psychophysiological onset insomnia in males: a randomized pilot study. Altern Ther Health Med; 19: 38-43. |
| A275 | Naidoo P, Pellow J (2013). A randomized placebo-controlled pilot study of Cat saliva 9cH and Histaminum 9cH in cat allergic adults. Homeopathy; 102: 123-129. |
| A276 | Pérol D, Provençal J, Hardy-Bessard AC, Coeffic D, Jacquin JP, Agostini C, Bachelot T, Guastalla JP, Pivot X, Martin JP, Bajard A, Ray-Coquard I (2012). Can treatment with Cocculine improve the control of chemotherapy-induced emesis in early breast cancer patients? A randomized, multi-centered, double-blind, placebo-controlled Phase III trial. BMC Cancer; 12: 603. |
| A277 | Razlog R, Pellow J, White SJ (2012). A pilot study on the efficacy of Valeriana officinalis mother tincture and Valeriana officinalis 3x in the treatment of attention deficit hyperactivity disorder. Health SA Gesondheid; 17; #603. |
| A278 | Sencer SF, Zhou T, Freedman LS, Ives JA, Chen Z, Wall D, Nieder ML, Grupp SA, Yu LC, Sahdev I, Jonas WB, Wallace JD, Oberbaum M (2012). Traumeel S in preventing and treating mucositis in young patients undergoing SCT: a report of the Children's Oncology Group. Bone Marrow Transplant; 47: 1409-1414. |
| A279 | Zanasi A, Mazzolini M, Tursi F, Morselli-Labate AM, Paccapelo A, Lecchi M (2013). Homeopathic medicine for acute cough in upper respiratory tract infections and acute bronchitis: A randomized, double-blind, placebo-controlled trial. Pulm Pharmacol Ther: In Press. |
| A280 | Liu L-L, Wan K-S, Cheng C-F, Tsai M-H, Wu Y-L, Wu W-F (2013). Effectiveness of MORA electronic homeopathic copies of remedies for allergic rhinitis: A short-term, randomized, placebo-controlled pilot study. Eur J Integr Med; 5: 119-125. |
| A281 | Mourão LC, Moutinho H, Canabarro A (2013). Additional benefits of homeopathy in the treatment of chronic periodontitis: A randomized clinical trial. Complement Ther Clin Pract; 19: 246-250. |
| A282 | Relton C, O'Cathain A, Nicholl J (2012). A pilot 'cohort multiple randomised controlled trial' of treatment by a homeopath for women with menopausal hot flushes. Contemp Clin Trials; 33: 853-859. |
| A283 | Sinha MN, Siddiqui VA, Nayak C, Singh V, Dixit R, Dewan D, Mishra A (2012). Randomized controlled pilot study to compare homeopathy and conventional therapy in acute otitis media. Homeopathy; 101: 5-12. |
| A284 | Teut M, Schnabel K, Baur R, Kerckhoff A, Reese F, Pilgram N, Berger F, Luedtke R, Witt CM (2013). Effects and feasibility of an Integrative Medicine program for geriatric patients - a cluster-randomized pilot study. Clin Interv Aging; 8: 953-961. |
| A285 | González de Vega C, Speed C, Wolfarth B, González J (2013). Traumeel vs. diclofenac for reducing pain and improving ankle mobility after acute ankle sprain: A multicentre, randomised, blinded, controlled and non-inferiority trial. Int J Clin Pract; 67: 979-989. |
| A286 | Pellow J, Swanepoel M (2013). A randomised pilot study on the efficacy of milking cream and a homeopathic complex topical cream on diaper dermatitis. Health SA Gesondheid; 18; #680. |
| A287 | Villanueva DFD, Rodríguez AP, García LRG, Osés CAM (2012). Use of homeopathic formula in malnourished children. Int J High Dilution Res; 11: 25-32. |
| A288 | Beer AM, Fey S, Zimmer M, Teske W, Schremmer D, Wiebelitz KR (2012). [Effectiveness and safety of a homeopathic drug combination in the treatment of chronic low back pain. A double-blind, randomized, placebo-controlled clinical trial]. MMW Fortschr Med; 154 (Suppl 2): 48-57. |
| A289 | Piraneo S, Maier J, Nervetti G, Duca P, Valli C, Milanesi A, Pagano F, Scaglione D, Osio M, Nascimbene C (2012). A randomized controlled clinical trial comparing the outcomes of homeopathic-phytotherapeutic and conventional therapy of whiplash in an emergency department. Homoeopathic Links; 25: 50-55. |
| A290 | Souza LM deA, Dantas ALL, Ribeiro A deO, Ramacciato JC, Motta, RHL (2012). Anti-edematous action: Arnica montana 6ch X diclofenac sodium 50 mg [Ação anti-edematosa: Arnica montana 6ch X diclofenaco de sódio 50 mg]. Pesq Bras Odontoped Clin Integr; 11: 491-496. |
| R227 | Chebel IFO (2012). Action of homeopathic treatment in the symptoms of burning mouth syndrome in two phases: double-blind placebo controlled study and open trial study. Thesis, University of São Paulo, Brazil. |
| R228 | Peckham EJ (2012). Is homeopathic treatment more effective than giving time and attention to NHS patients with chronic complaints. Thesis, University of Leeds, UK. |
| R229 | Macrì F, Moretti M, Massaccesi V, Peparini I, Lambiase C, Tromba V (2012). Effect of Apis Mellifica on histamine skin test itchy swelling. Eur J Integr Med; 4 (Suppl 1): 69. |
| R230 | Sharma N, Sharma S, Shekhawat U (2012). Effect of homeopathy in women with heavy menstrual bleeding. BJOG; 119 (Suppl s1): 195-196. |
| R231 | Sharma S, Sharma N, Sharma R (2012). Accelerating the healing of bone fracture using homeopathy: a prospective, randomized double-blind controlled study. BMC Complement Altern Med; 12(Suppl 1): O61. |
| R232 | Sharma S, Sharma N (2012). Long term evaluation of homeopathy on post treatment impairment of pulmonary tuberculosis. BMC Complement Altern Med; 12(Suppl 1): P223. |
| R233 | Sharma S, Sharma N, Sharma CM (2012). A randomized, double-blind, placebo controlled crossover trial of homeopathy in patients with chronic malignant pain. Pain Pract 12 (Suppl 1): 53. |
| R234 | Sharma N, Sharma S, Sharma S (2013). Individualized homeopathy as an adjuvant in treatment for hypertension in older adults: Results from randomized double blind placebo controlled trial. Forsch Komplementmed; 20(suppl 1): 38-39. |
| R235 | Sharma N, Ameta A, Sharma S (2013). Effect of homeopathy on chronic tension-type headache: a pragmatic, randomised controlled single blind trial. J Headache Pain; 14 (Suppl 1): P56. |
| R236 | von Ammon K, Sauter U, Frei H, Kretschmar S, Thurneysen A, Frei-Erb M (2012). Classical homeopathy helps hyperactive children – a 10-year follow-up of homeopathic and integrated medical treatment in children suffering from attention deficit disorder with and without hyperactivity. Eur J Integr Med; 4 (Suppl 1): 73-74. |
| R237 | von Ammon K, Sauter U, Kretschmar S, Frei H, Thurneysen, A, Frei-Erb, M (2013). Long-term effects of homeopathic treatment in children suffering from attention deficit disorder with and without hyperactivity. Int J High Dilution Res; 12: 119-120. |
| R238 | Oberai P, Varanasi R, Gopinadhan S, Mishra A, Singh V, Nayak C (2013). Homoeopathic management of attention deficit hyperactivity disorder: A randomised placebo controlled pilot trial. Forsch Komplementmed; 20(suppl 1): 353. |
| R239 | Kundu T, Shaikh A, Kutty A, Nalvade A, Kulkarni S, Kulkarni R, Ghosh K (2012). Homeopathic medicines substantially reduce the need for clotting factor concentrates in haemophilia patients: Results of a blinded placebo controlled cross over trial. Homeopathy; 101: 38-43. |
| R240 | Saeed-ul-Hassan S, Tariq I, Khalid A, Karim S (2013). Comparative clinical study on the effectiveness of homeopathic combination remedy with standard maintenance therapy for Dengue fever. Trop J Pharm Res; 12: 767-770. |
| R241 | Hellhammer J, Schubert M (2013). Effects of a homeopathic combination remedy on the acute stress response, well-being, and sleep: a double-blind, randomized clinical trial. J Altern Complement Med; 19: 161-169. |
| R242 | D'Souza RP (2012). Efficacy of homeopathy in treatment of acute otitis media with intense otalgia in children. Homoeopathic Heritage International; 37: 37-42. |
| R243 | Verma DK, Verma SK, Atray JP, Atray M (2013). Homeopathy and epidemic viral conjunctivitis: A single blind study. Homoeopathic Links; 26: 58-62. |

The category ‘peer reviewed’ now includes any paper published in the *Indian Journal of Research in Homoeopathy* from the start of 2013, when the journal became fully peer reviewed – *Indian J Res Homoeopathy* 2013; **7**: 34–37.
