# Supplementary material for: Randomised placebo-controlled trials of individualised homeopathic treatment: systematic review and meta-analysis
Source: Syst Rev. 2014 Dec 6;3:142. doi: 10.1186/2046-4053-3-142 (PMC4326322; doi:10.1186/2046-4053-3-142)
Supplement: Supplementary file 3 — Additional file 3: PRISMA flowchart for all records published up to and including 2013. (DOC 58 KB) [file 13643_2014_328_MOESM3_ESM.doc]

**Additional file**
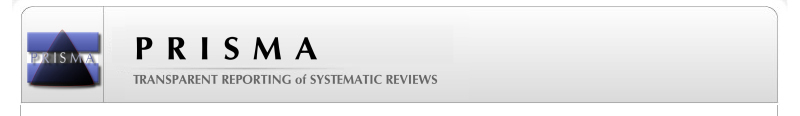
 **3: *PRISMA* 2009 Flow Diagram**

**Screening**

**Included**

**Eligibility**

**Identification**

Records identified through database searching
(n = **456**)

Additional records identified through other sources
(n = **77**)

Records after duplicates removed
(n = **437**)

Records screened
(n = **437**)

Records excluded
(n = **89**)

Full-text records assessed for eligibility
(n = **348**)

Full-text records excluded, with reasons
(n = **317**)

Not peer-reviewed: **128**

Not randomised and/or not controlled: **20**

Not ‘homeopathic’: **12**

Not placebo-controlled or individualised: **142**

Not eligible study design/outcome: **15**

Records included in qualitative synthesis
(n = **31**)

Records included in quantitative synthesis (meta-analysis)
(n = **21**)
