# Supplementary material for: Randomised placebo-controlled trials of individualised homeopathic treatment: systematic review and meta-analysis
Source: Syst Rev. 2014 Dec 6;3:142. doi: 10.1186/2046-4053-3-142 (PMC4326322; doi:10.1186/2046-4053-3-142)
Supplement: Supplementary file 4 — Additional file 4: Details of records of RCTs of individualised homeopathy. Asterisk: paper reports two RCTs. Double asterisk: trial reported as double-blinded, but revealed to be single-blinded on inspection of published protocol. SD standard deviation. Reference numbering continues from previously published listings [11]. (DOCX 60 KB) [file 13643_2014_328_MOESM4_ESM.docx]

**Additional file 4**

**15 records excluded from systematic review**:

**Crossover trials**:

A8: Carlini

A12: Fisher

A15: Frei

A27: Kuzeff

A28: Lökken

**Single-blinded trials**:

A17: Haila

A29: Mousavi

A264: Adler

A265: Chakraborti

A266: Chakraborti

A267: Oberai

**A268: Saha

**Secondary outcomes only**:

A2: Bell

A3: Bell

A4: Bell

**5 records (as per search update for 2012-2013)**:

**A264 – A268**

**41 records (as per original**

**literature search)**:

**A1 – A41**

**31 records included in systematic review**

**21 records included in meta-analysis**:

A5: Bell

A6: Bonne

A7: Brien

A9: Cavalcanti

A10: Chapman

A11: de Lange de Klerk

A13: Fisher

A14: Frass

A19: Jacobs

A20: Jacobs

A22: Jacobs

A23: Jacobs

A25: Kainz

*A31: Rastogi

A32: Sajedi

A33: Siebenwirth

A35: Straumsheim

A36: Thompson

A38: Weatherley-Jones

A40: Whitmarsh

A41: Yakir

**10 records excluded from meta-analysis**:

***Data not provided****:*

A16: Gaucher

A21: Jacobs

A24: Jansen

***Group SDs not derivable****:*

A1: Andrade

A18: Jacobs

A26: Katz

A30: Naudé

A39: White

***Non-parametric data****:*

A34: Steinsbekk

A37: Walach

* Reports **two** RCTs. ** Reported as double-blinded, but revealed to be *single*-blinded on inspection of published protocol. SD: Standard Deviation.

*Reference numbering continues from previously published listings.*^11^

**References for Additional file 4**:

A1 Andrade L, Ferraz MB, Atra E, Castro A, Silva MSM (1991). A randomized controlled trial to evaluate the effectiveness of homoeopathy in rheumatoid arthritis. *Scandinavian Journal of Rheumatology*; **20**: 204–208.

A2 Bell IR, Lewis DA 2nd, Brooks AJ, Schwartz GE, Lewis SE, Caspi O, Cunningham V, Baldwin CM (2004). Individual differences in response to randomly assigned active individualized homeopathic and placebo treatment in fibromyalgia: implications of a double-blinded optional crossover design. *Journal of Alternative & Complementary Medicine*; **10**: 269–283.

A3 Bell IR, Lewis DA 2nd, Schwartz GE, Lewis SE, Caspi O, Scott A, Brooks AJ, Baldwin CM (2004). Electroencephalographic cordance patterns distinguish exceptional clinical responders with fibromyalgia to individualized homeopathic medicines*. Journal of Alternative & Complementary Medicine*; **10**: 285–299.

A4 Bell IR, Lewis DA 2nd, Lewis SE, Schwartz GE, Brooks AJ, Scott A, Baldwin CM (2004). EEG alpha sensitization in individualized homeopathic treatment of fibromyalgia*. International Journal of Neuroscience*; **114:** 1195–1220.

A5 Bell I, Lewis D, Brooks A, Schwartz G, Lewis S, Walsh B, Baldwin C (2004). Improved clinical status in fibromyalgia patients treated with individualized homeopathic remedies versus placebo. *Rheumatology*; **43**: 577–582.

A6 Bonne O, Shemer Y, Gorali Y, Katz M, Shalev AY (2003). A randomized, double-blind, placebo-controlled study of classical homeopathy in generalized anxiety disorder. *Journal of Clinical Psychiatry*; **64**: 282–287.

A7 Brien S, Lachance L, Prescott P, McDermott C, Lewith G (2011). Homeopathy has clinical benefits in rheumatoid arthritis patients that are attributable to the consultation process but not the homeopathic remedy: a randomized controlled clinical trial. *Rheumatology* (Oxford); **50**: 1070-1082.

A8 Carlini EA, Braz S, Troncone LRP, Tufik S, Romanach AK, Pustiglione M, Sposati MC, Cudizio Filho O, Prado MIA (1987). Efeito hipnótico de medicação homeopática e do placebo. Avaliação pela técnica de duplo-cego e cruzamento [Hypnotic effect of homeopathic medication and placebo. Evaluation by double-blind and crossover techniques]. Revista *Da Associação Médica Brasileira*; **33**: 83–88.

A9 Cavalcanti AM, Rocha LM, Carillo R Jr, Lima LU, Lugon JR (2003). Effects of homeopathic treatment on pruritus of haemodialysis patients: a randomized placebo-controlled double-blind trial. *Homeopathy*; **92**: 177–181.

A10 Chapman EH, Weintraub RJ, Milburn MA, Pirozzi TO, Woo E (1999). Homeopathic treatment of mild traumatic brain injury: a randomized, double-blind, placebo-controlled clinical trial. *Journal of Head Trauma Rehabilitation*; **14**: 521–542.

A11 de Lange de Klerk ESM, Blommers J, Kuik DJ, Bezemer PD, Feenstra L (1994). Effects of homoeopathic medicines on daily burden of symptoms in children with recurrent upper respiratory tract infections. *British Medical Journal*; **309**: 1329–1332.

A12 Fisher P, Scott DL (2001). A randomized controlled trial of homeopathy in rheumatoid arthritis. *Rheumatology*; **40**: 1052–1055.

A13 Fisher P, McCarney R, Hasford C, Vickers A (2006). Evaluation of specific and non-specific effects in homeopathy: Feasibility study for a randomised trial. *Homeopathy*; **95**: 215–222.

A14 Frass M, Linkesch M, Banyai S, Resch G, Dielacher C, Lobl T, Endler C, Haidvogl M, Muchitsch I, Schuster E (2005). Adjunctive homeopathic treatment in patients with severe sepsis: a randomized, double-blind, placebo-controlled trial in an intensive care unit. *Homeopathy*; **94**: 75–80.

A15 Frei H, Everts R, von Ammon K, Kaufmann F, Walther D, Hsu-Schmitz SF, Collenberg M, Fuhrer K, Hassink R, Steinlin M, Thurneysen A (2005). Homeopathic treatment of children with attention deficit hyperactivity disorder: a randomized, double blind, placebo controlled crossover trial. *European Journal of Pediatrics*; **164**: 758–767.

A16 Gaucher C, Jeulin D, Peycru P, Amengual C (1994). A double blind randomized placebo controlled study of cholera treatment with highly diluted and succussed solutions. *British Homoeopathic Journal*; **83**: 132-134.

A17 Haila S, Koskinen A, Tenovuo J (2005). Effects of homeopathic treatment on salivary flow rate and subjective symptoms in patients with oral dryness: a randomized trial. *Homeopathy*; **94**: 175–181.

A18 Jacobs J, Jiminez LM, Gloyds SS, Casares FE, Gaitan MP, Crothers D (1993). Homoeopathic treatment of acute childhood diarrhoea. A randomized clinical trial in Nicaragua. *British Homoeopathic Journal*; **82**: 83–86.

A19 Jacobs J, Jimenez LM, Gloyds SS, Gale JL, Crothers D (1994). Treatment of acute childhood diarrhea with homeopathic medicine; a randomized clinical trial in Nicaragua. *Pediatrics*; **93**: 719–725.

A20 Jacobs J, Springer DA, Crothers D (2001). Homeopathic treatment of acute otitis media in children: a preliminary randomized placebo-controlled trial. *Pediatric Infectious Disease Journal*; **20**: 177–183.

A21 Jacobs J, Jimenez LM, Malthouse S, Chapman E, Crothers D, Masuk M, Jonas WB (2000). Homeopathic treatment of acute childhood diarrhoea: results from a clinical trial in Nepal. *Journal of Alternative and Complementary Medicine*; **6**: 131–139.

A22 Jacobs J, Herman P, Heron K, Olsen S, Vaughters L (2005b). Homeopathy for menopausal symptoms in breast cancer survivors: a preliminary randomized controlled trial. *Journal of Alternative and Complementary Medicine*; **11**: 21–27.

A23 Jacobs J, Williams A-L, Girard C, Njike VY, Katz D (2005a). Homeopathy for attention-deficit/hyperactivity disorder: a pilot randomized-controlled trial. *Journal of Alternative and Complementary Medicine*; **11**: 799–806.

A24 Jansen GRHJ, van der Veer ALJ, Hagenaars J, van der Juy A (1992). Lessons learnt from an unsuccessful clinical trial of homoeopathy. Results of a small-scale, double-blind trial in proctocolitis. *British Homoeopathic Journal*; **81**: 132–138.

A25 Kainz JT, Kozel G, Haidvogl M, Smolle J (1996). Homoeopathic versus placebo therapy of children with warts on the hands: a randomized, double-blind clinical trial. *Dermatology*; **193**: 318–320.

A26 Katz T, Fisher P, Katz A, Davidson J, Feder G (2005). The feasibility of a randomised, placebo-controlled clinical trial of homeopathic treatment of depression in general practice. *Homeopathy*; **94**: 145-52.

A27 Kuzeff RM (1998). Homeopathy, sensation of well-being and CD4-levels – A placebo-controlled, randomized trial. *Complementary Therapies in Medicine*; **6**: 4–9.

A28 Lökken P, Straumsheim PA, Tveiten D, Skjelbred P, Borchgrevink CF (1995). Effect of homoeopathy on pain and other events after acute trauma; placebo controlled trial with bilateral oral surgery. *British Medical Journal*; **310**: 1439–1442.

A29 Mousavi F, Mojaver YN, Asadzadeh M, Mirzazadeh M (2009). Homeopathic treatment of minor aphthous ulcer: a randomized, placebo-controlled clinical trial. *Homeopathy*; **98**: 137-141.

A30 Naudé DF, Couchman IMS, Maharaj A (2010). Chronic primary insomnia: efficacy of homeopathic simillimum. Homeopathy; **99**: 63–68. [Published erratum: *Homeopathy*; 2010; **99**: 151]

A31 Rastogi DP, Singh VP, Singh V, Dey SK, Rao K (1999). Homeopathy in HIV infection: a trial report of double-blind placebo controlled study*. British Homoeopathic Journal*; **88**: 49–57.

A32 Sajedi F, Alizad V, Alaeddini F, Fatemi R, Mazaherinezhad A (2008). The effect of adding homeopathic treatment to rehabilitation on muscle tone of children with spastic cerebral palsy. *Complementary Therapies in Clinical Practice*; **14**: 33–37.

A33 Siebenwirth J, Lüdtke R, Remy W, Rakoski J, Borelli S, Ring J (2009). Wirksamkeit einer klassisch-homöopathischen Therapie bei atopischem Ekzem. Eine randomisierte, placebokontrollierte Doppelblindstudie [Effectiveness of classical homeopathic treatment in atopic eczema. A randomised placebo-controlled double-blind clinical trial]. *Forschende Komplementärmedizin*; **16**: 315–323.

A34 Steinsbekk A, Bentzen N, Fønnebø V, Lewith G (2005). Self treatment with one of three self selected, ultramolecular homeopathic medicines for the prevention of upper respiratory tract infections in children. A double-blind randomized placebo controlled trial. *British Journal of Clinical Pharmacology*; **59**: 447–455.

A35 Straumsheim P, Borchgrevink C, Mowinckel P, Kierulf H, Hafslund O (2000). Homeopathic treatment of migraine: a double blind, placebo controlled trial of 68 patients. *British Homeopathic Journal*; **89**: 4–7.

A36 Thompson EA, Montgomery A, Douglas D, Reilly D (2005). A pilot, randomized, double-blinded, placebo-controlled trial of individualized homeopathy for symptoms of estrogen withdrawal in breast-cancer survivors. *Journal of Alternative and Complementary Medicine*; **11**: 13–20.

A37 Walach H, Häusler W, Lowes T, Mussbach D, Schamell U, Springer W, Stritzl G, Haag G (1997). Classical homeopathic treatment of chronic headaches*. Cephalalgia*; **17:** 119–126.

A38 Weatherley-Jones E, Nicholl JP, Thomas KJ, Parry GJ, McKendrick MW, Green ST, Stanley PJ, Lynch SP (2004). A randomized, controlled, triple-blind trial of the efficacy of homeopathic treatment for chronic fatigue syndrome. *Journal of Psychosomatic Research*; **56**: 189–197.

A39 White A, Slade P, Hunt C, Hart A, Ernst E (2003). Individualised homeopathy as an adjunct in the treatment of childhood asthma: a randomised placebo controlled trial. *Thorax*; **58**: 317–321.

A40 Whitmarsh TE, Coleston-Shields DM, Steiner TJ (1997). Double-blind randomized placebo-controlled study of homoeopathic prophylaxis of migraine. *Cephalalgia*; **17**: 600–604.

A41 Yakir M, Kreitler S, Brzezinski A, Vithoulkas G, Oberbaum M, Bentwich Z (2001). Effects of homeopathic treatment in women with premenstrual syndrome: a pilot study. *British Homeopathic Journal*; **90**: 148–153.

A264 Adler UC, Krüger S, Teut M, Lüdtke R, Schützler L, Martins F, Willich SN, Linde K, Witt CM (2013). Homeopathy for depression: A randomized, partially double-blind, placebo-controlled, four-armed study (DEP-HOM). *PLoS ONE*; **8**: e74537.

A265 Chakraborty PS, Lamba CD, Nayak D, John MD, Sarkar DB, Poddar A, Arya JS, Raju K, Vivekanand K, Singh HBK, Baig H, Prusty AK, Singh V, Nayak C (2013). Effect of individualized homoeopathic treatment in influenza like illness: A multicenter, single blind, randomized, placebo controlled study. *Indian J Res Homoeopathy*; **7**: 22–30.

A266 Chakraborty PS, Varanasi R, Majumdar AK, Banoth K, Prasad S, Ghosh MS, Sinha MN, Reddy GRC, Singh V, Nayak, C (2013). Effect of homoeopathic LM potencies in acute attacks of haemorrhoidal disease: A multicentric randomized single-blind placebo-controlled trial. *Indian J Res Homoeopathy*; **7**: 72–80.

A267 Oberai P, Gopinadhan S, Varanasi R, Mishra A, Singh V, Nayak C (2013). Homoeopathic management of attention deficit hyperactivity disorder: A randomised placebo-controlled pilot trial. *Indian J Res Homoeopathy*; **7**: 158–167.

A268 Saha S, Koley M, Hossain SI, Mundle M, Ghosh S, Nag G, Datta AK, Rath P (2013). Individualized homoeopathy versus placebo in essential hypertension: A double-blind randomized controlled trial. *Indian J Res Homoeopathy*; **7**: 62–71.
