# Supplementary material for: Randomised placebo-controlled trials of individualised homeopathic treatment: systematic review and meta-analysis
Source: Syst Rev. 2014 Dec 6;3:142. doi: 10.1186/2046-4053-3-142 (PMC4326322; doi:10.1186/2046-4053-3-142)
Supplement: Supplementary file 6 — Additional file 6: Data extracted for meta-analysis of RCTs with: (a) continuous main outcome measure and (b) dichotomous main outcome measure. (DOCX 19 KB) [file 13643_2014_328_MOESM6_ESM.docx]

| **Study** | **Homeopathy** | | | **Placebo** | | |
| --- | --- | --- | --- | --- | --- | --- |
|  | **Mean** | **SD** | **Total** | **Mean** | **SD** | **Total** |
| Andrade 1991 | 0.0 | 0.0 | 0 | 0.0 | 0.0 | 0 |
| Jacobs 1993 | 0.0 | 0.0 | 0 | 0.0 | 0.0 | 0 |
| de Lange de Klerk 1994 | 2.21 | 1.41 | 86 | 2.61 | 1.41 | 84 |
| Jacobs 1994 | 3.0 | 1.9 | 40 | 3.8 | 1.7 | 41 |
| Walach 1997 | 0.0 | 0.0 | 0 | 0.0 | 0.0 | 0 |
| Whitmarsh 1997 | 2.7 | 1.23 | 30 | 3.4 | 3.01 | 30 |
| Chapman 1999 | 2.61 | 0.65 | 27 | 2.87 | 0.71 | 23 |
| Rastogi(a) 1999 | 580.21 | 243.9 | 19 | 622.61 | 246.5 | 23 |
| Rastogi(b) 1999 | 534.35 | 278.23 | 20 | 452.0 | 158.67 | 18 |
| Jacobs 2000 | 0.0 | 0.0 | 0 | 0.0 | 0.0 | 0 |
| Straumsheim 2000 | 3.5 | 2.6 | 35 | 3.2 | 2.2 | 33 |
| Yakir 2001 | 0.13 | 0.12 | 11 | 0.34 | 0.3 | 8 |
| Bonne 2003 | 21.7 | 11.6 | 20 | 20.9 | 9.2 | 19 |
| White 2003 | 0.0 | 0.0 | 0 | 0.0 | 0.0 | 0 |
| Bell 2004 | 71.3 | 36.3 | 26 | 82.8 | 36.0 | 27 |
| Jacobs 2005a | 62.65 | 14.96 | 20 | 60.88 | 12.07 | 17 |
| Jacobs 2005b | 48.3 | 10.0 | 18 | 56.2 | 10.86 | 15 |
| Katz 2005 | 0.0 | 0.0 | 0 | 0.0 | 0.0 | 0 |
| Steinsbekk 2005 | 0.0 | 0.0 | 0 | 0.0 | 0.0 | 0 |
| Thompson 2005 | 2.9 | 0.9 | 23 | 3.2 | 0.7 | 22 |
| Fisher 2006 | 3.51 | 1.99 | 15 | 3.83 | 1.9 | 12 |
| Sajedi 2008 | 2.5 | 0.9 | 7 | 2.1 | 1.3 | 9 |
| Siebenwirth 2009 | 40.7 | 12.5 | 5 | 32.7 | 21.8 | 9 |
| Naudé 2010 | 0.0 | 0.0 | 0 | 0.0 | 0.0 | 0 |

**Additional file 6 (a): Trials with continuous main outcome measure**

**Additional file 6 (b): Trials with dichotomous main outcome measure**

| **Study** | **Homeopathy** | | **Placebo** | |
| --- | --- | --- | --- | --- |
|  | **Events** | **Total** | **Events** | **Total** |
| Jansen 1992 | 0 | 0 | 0 | 0 |
| Gaucher 1994 | 0 | 0 | 0 | 0 |
| Kainz 1996 | 9 | 30 | 7 | 30 |
| Jacobs 2001 | 29 | 36 | 27 | 39 |
| Cavalcanti 2003 | 7 | 11 | 3 | 9 |
| Weatherley-Jones 2004 | 20 | 43 | 16 | 43 |
| Frass 2005 | 25 | 33 | 17 | 34 |
| Brien 2010 | 5 | 12 | 5 | 11 |

Trials are arranged chronologically within their category of main outcome measure
