# Supplementary figures and images for: Randomised placebo-controlled trials of individualised homeopathic treatment: systematic review and meta-analysis
Source: Syst Rev. 2014 Dec 6;3:142. doi: 10.1186/2046-4053-3-142 (PMC4326322; doi:10.1186/2046-4053-3-142)

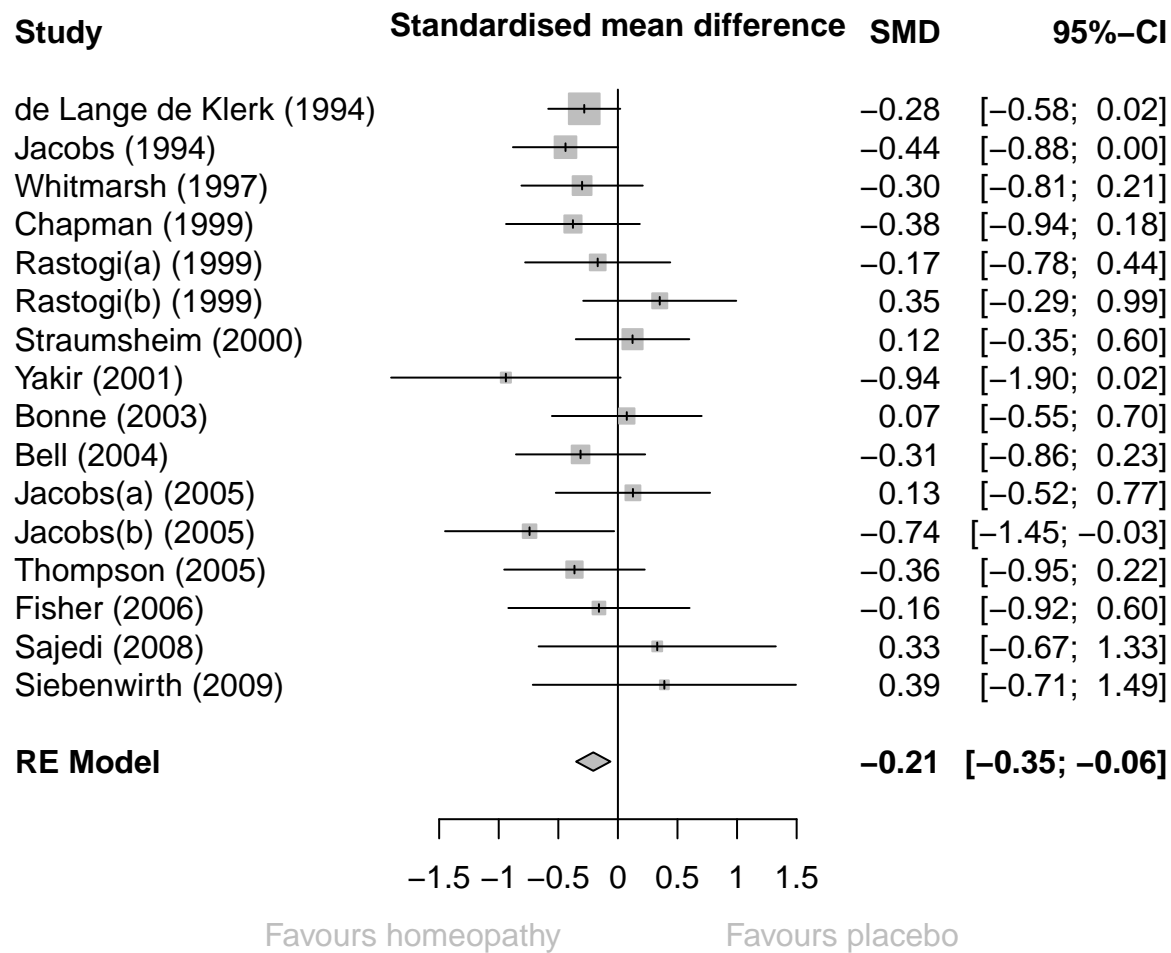

Supplement: Supplementary file 7 — Additional file 7: Standardised mean difference (SMD) and 95% confidence interval (CI) for RCTs with continuous main outcome measure, showing pooled statistic (random effects [RE] model). (PDF 6 KB) [file 13643_2014_328_MOESM7_ESM.pdf]
