# Supplementary material for: Randomised placebo-controlled trials of individualised homeopathic treatment: systematic review and meta-analysis
Source: Syst Rev. 2014 Dec 6;3:142. doi: 10.1186/2046-4053-3-142 (PMC4326322; doi:10.1186/2046-4053-3-142)
Supplement: Supplementary file 8 — Additional file 8: Sensitivity analysis on risk-of-bias rating, and including specified demographic data per trial. (DOCX 24 KB) [file 13643_2014_328_MOESM8_ESM.docx]

**Additional file 8**

| # | **First author** | **Year** | **Risk-of-bias rating** | **In previous meta-analysis?** | **Imputed data?** | **OR [95%CI] per trial** | **Total sample per trial** | **Pooled OR [95% CI] for N trials** | **Total sample for N trials** | **N trials included** | ***P* for N trials** |
| --- | --- | --- | --- | --- | --- | --- | --- | --- | --- | --- | --- |
| A05 | **Bell** | 2004 | **B1** * | N | N | 1.77 [0.66, 4.72] | 53 | 1.98 [1.16, 3.38] | 209 | 3 | 0.013 |
| A19 | **Jacobs** | 1994 | **B1** * | Y | N | 2.22 [1.00, 4.94] | 81 |  |  |  |  |
| A20 | **Jacobs** | 2001 | **B1** * | Y | N | 1.84 [0.63, 5.36] | 75 |  |  |  |  |
| A23 | **Jacobs** | 2005a | **B1** | N | N | 0.80 [0.25, 2.57] | 37 | 1.77 [1.18, 2.66] | 341 | 6 | 0.006 |
| A10 | **Chapman** | 1999 | **B1** | Y | N | 1.98 [0.72, 5.49] | 50 |  |  |  |  |
| A36 | **Thompson** | 2005 | **B1** | N | Y | 1.94 [0.66, 5.64] | 45 |  |  |  |  |
| A35 | **Straumsheim** | 2000 | **B2** | Y | Y | 0.80 [0.34, 1.90] | 68 | 1.66 [1.17, 2.34] | 476 | 8 | 0.005 |
| A14 | **Frass** | 2005 | **B2** | N | N | 3.13 [1.10, 8.86] | 67 |  |  |  |  |
| A06 | **Bonne** | 2003 | **B3** | N | N | 0.87 [0.28, 2.73] | 39 | 1.64 [1.17, 2.29] | 534 | 10 | 0.004 |
| A41 | **Yakir** | 2001 | **B3** | Y | N | 5.50 [0.96, 31.62] | 19 |  |  |  |  |
| A11 | **de Lange de Klerk** | 1994 | **B4** | Y | Y | 1.67 [0.96, 2.89] | 170 | 1.65 [1.24, 2.18] | 704 | 11 | < 0.001 |
| A25 | **Kainz** | 1996 | **B6** | Y | N | 1.41 [0.45, 4.45] | 60 | 1.63 [1.24, 2.14] | 764 | 12 | < 0.001 |
| A22 | **Jacobs** | 2005b | **C1.0** | N | Y | 3.84 [1.06, 13.90] | 33 | 1.66 [1.28, 2.17] | 820 | 14 | < 0.001 |
| A07 | **Brien** | 2011 | **C1.0** | N | Y | 0.86 [0.16, 4.47] | 23 |  |  |  |  |
| A38 | **Weatherley-Jones** | 2004 | **C1.1** | N | N | 1.47 [0.62, 3.47] | 86 | 1.65 [1.28, 2.12] | 906 | 15 | < 0.001 |
| A13 | **Fisher** | 2006 | **C1.2** | N | N | 1.33 [0.34, 5.30] | 27 | 1.64 [1.28, 2.09] | 933 | 16 | < 0.001 |
| A09 | **Cavalcanti** | 2003 | **C1.3** | N | Y | 3.50 [0.55, 22.30] | 20 | 1.66 [1.30, 2.12] | 953 | 17 | < 0.001 |
| A40 | **Whitmarsh** | 1997 | **C1.4** | Y | Y | 1.72 [0.69, 4.34] | 60 | 1.63 [1.29, 2.06] | 1029 | 19 | < 0.001 |
| A32 | **Sajedi** | 2008 | **C1.4** | N | N | 0.55 [0.09, 3.34] | 16 |  |  |  |  |
| A33 | **Siebenwirth** | 2009 | **C2.1** | N | Y | 0.49 [0.07, 3.65] | 14 | 1.60 [1.27, 2.03] | 1043 | 20 | < 0.001 |
| A31 | **Rastogi (a)** | 1999 | **C2.5** | N | N | 1.36 [0.45, 4.10] | 42 | 1.53 [1.22, 1.91] | 1123 | 22 | < 0.001 |
| A31 | **Rastogi (b)** | 1999 | **C2.5** | N | N | 0.53 [0.17, 1.69] | 38 |  |  |  |  |

* Reliable evidence. Y, Yes; N, No.

*‘Effect size’ was interpreted based on Standardised Mean Difference (SMD) as follows: SMD <0.40 = ‘small’; SMD 0.40 to 0.70 = ‘moderate’; SMD >0.70 = ‘large’:* Schünemann HJ, Oxman AD, Vist GE, et al. Chapter 12: Interpreting results and drawing conclusions. In: Higgins JPT, Green S (eds). *Cochrane Handbook for Systematic Reviews of Interventions; Version 5.1.0.* The Cochrane Collaboration, 2011.

*Using the standard formula to convert SMD approximately to OR, our corresponding ‘effect size’ thresholds were calculated to be: OR <2.10 = ‘small’; OR 2.10 to 3.60 = ‘moderate’; OR >3.60 = ‘large’.*
