# Supplementary material for: Randomised placebo-controlled trials of individualised homeopathic treatment: systematic review and meta-analysis
Source: Syst Rev. 2014 Dec 6;3:142. doi: 10.1186/2046-4053-3-142 (PMC4326322; doi:10.1186/2046-4053-3-142)
Supplement: Supplementary file 9 — Additional file 9: Meta-analysed trials in common with those included by Shang et al. [8] , showing comparison of quality assessment and degree of similarity of selected outcome measure. (DOCX 14 KB) [file 13643_2014_328_MOESM9_ESM.docx]

**Additional file 9**

| **First author (date)** | **Trial ID – Mathie** | **Trial ID – Shang** | **Risk-of-bias rating – Mathie** | **‘High quality’ – Shang** | **Same outcome measure** |
| --- | --- | --- | --- | --- | --- |
| de Lange de Klerk (1994) | A11 | 27 | B4 | No | Yes |
| Jacobs (1994) | A19 | 49 | B1* | Yes | Yes |
| Kainz (1996) | A25 | 52 | B6 | No | Yes |
| Whitmarsh (1997) | A40 | 99 | C1.4 | No | No |
| Chapman (1999) | A10 | 21 | B1 | Yes | No |
| Straumsheim (2000) | A35 | 87 | B2 | No | No |
| Jacobs (2001) | A20 | 47 | B1* | Yes | Yes |
| Yakir (2001) | A41 | 104 | B3 | No | Yes |

* Reliable evidence

Details from Shang taken from tabulation and forest plot, posted on authors’ website, 23 December 2005: <http://www.ispm.ch/fileadmin/doc_download/1433.Study_characteristics_of_homoeopathy_studies_corrected.pdf> (accessed, 12 May 2014).
