# Supplementary material for: Randomised placebo-controlled trials of individualised homeopathic treatment: systematic review and meta-analysis
Source: Syst Rev. 2014 Dec 6;3:142. doi: 10.1186/2046-4053-3-142 (PMC4326322; doi:10.1186/2046-4053-3-142)
Supplement: Supplementary file 10 — Additional file 10: Sub-group analysis showing interaction for: (a) all N = 22 trials with analysable data, (b) N = 12 (‘B’-rated) trials with uncertain risk of bias and (c) sub-set of (‘B1’-rated) trials with reliable evidence. (DOCX 22 KB) [file 13643_2014_328_MOESM10_ESM.docx]

|  |  |  |  |  |  |  |  | **(a) All trials with analysable data: N=22** | | | | |
| --- | --- | --- | --- | --- | --- | --- | --- | --- | --- | --- | --- | --- |
|  |  |  |  |  |  |  |  | **'Yes'** | | **'No'** | | ***P* for interaction between sub-groups** |
|  |  |  |  |  |  |  |  | **Pooled OR** | **N** | **Pooled OR** | **N** |  |
| Study included in previous ‘global’ meta-analyses of homeopathy? | | | | | | | | 1.67 [1.22, 2.28] | 8 | 1.39 [1.01, 1.92] | 14 | 0.42 |
| Pilot (feasibility) study? | | | | | | | | 1.87 [1.24, 2.82] | 8 | 1.40 [1.07, 1.84] | 14 | 0.25 |
| Sample size > median for N=22 trials? | | | | | | | | 1.69 [1.30, 2.21] | 11 | 1.20 [0.76, 1.89] | 11 | 0.20 |
| Potency of homeopathic medicines > 12C? | | | | | | | | 2.00 [1.38, 2.88] | 8 | 1.30 [0.98, 1.73] | 14 | 0.071 |
| Data for meta-analysis were imputed? | | | | | | | | 1.54 [1.09, 2.19] | 8 | 1.52 [1.13, 2.04] | 14 | 0.95 |
| Free of vested interest? | | | | | | | | 1.57 [1.05, 2.36] | 4 | 1.51 [1.14, 2.00] | 18 | 0.87 |
|  |  |  |  |  |  |  |  |  |  |  |  |  |
|  |  |  |  |  |  |  |  | **(b) Trials with uncertain risk of bias: N=12** | | | | |
|  |  |  |  |  |  |  |  | **'Yes'** | | **'No'** | | ***P* for interaction between sub-groups** |
|  |  |  |  |  |  |  |  | **Pooled OR** | **N** | **Pooled OR** | **N** |  |
| Study included in previous ‘global’ meta-analyses of homeopathy? | | | | | | | | 1.66 [1.20, 2.32] | 7 | 1.56 [0.96, 2.56] | 5 | 0.84 |
| Pilot (feasibility) study? | | | | | | | | 1.77 [1.12, 2.80] | 6 | 1.55 [1.06, 2.26] | 6 | 0.65 |
| Sample size > median for N=22 trials? | | | | | | | | 1.30 [0.46, 3.66] | 3 | 1.72 [1.28, 2.31] | 9 | 0.83 |
| Potency of homeopathic medicines > 12C? | | | | | | | | 2.05 [1.38, 3.06] | 7 | 1.33 [0.92, 1.94] | 5 | 0.12 |
| Data for meta-analysis were imputed? | | | | | | | | 1.41 [0.87, 2.27] | 3 | 1.79 [1.26, 2.55] | 9 | 0.43 |
| Free of vested interest? | | | | | | | | 1.69 [1.05, 2.73] | 2 | 1.61 [1.14, 2.26] | 10 | 0.86 |
|  |  |  |  |  |  |  |  |  |  |  |  |  |
|  |  |  |  |  |  |  |  | **(c) Sub-set of trials with reliable evidence: N=3** | | | | |
|  |  |  |  |  |  |  |  | **'Yes'** | | **'No'** | | ***P* for interaction between sub-groups** |
|  |  |  |  |  |  |  |  | **Pooled OR** | **N** | **Pooled OR** | **N** |  |
| Study included in previous ‘global’ meta-analyses of homeopathy? | | | | | | | | 2.08 [1.09, 3.94] | 2 | 1.77 [0.66, 4.72] | 1 | 0.79 |
| Pilot (feasibility) study? | | | | | | | | 1.80 [0.87, 3.71] | 2 | 2.22 [1.00, 4.94] | 1 | 0.70 |
| Sample size > median for N=22 trials? | | | | | | | | 1.97 [1.16, 3.38] | 3 |  |  |  |
| Potency of homeopathic medicines > 12C? | | | | | | | | 2.08 [1.09, 3.94] | 2 | 1.77 [0.66, 4.72] | 1 | 0.79 |
| Data for meta-analysis were imputed? | | | | | | | | - | - | 1.97 [1.16, 3.38] | 3 |  |
| Free of vested interest? | | | | | | | | 1.77 [0.66, 4.72] | 1 | 2.08 [1.09, 3.94] | 2 | 0.79 |

Odds ratio (OR) values shown in red are those with *non-significant* effect favouring homeopathy

‘No’ for ‘Potency of homeopathic medicines > 12C’ includes trials with either mixed or unknown potencies

‘No’ for ‘Free of vested interest’ includes trials where assessment was ‘unclear’
