# Supplementary material for: Randomised placebo-controlled trials of individualised homeopathic treatment: systematic review and meta-analysis
Source: Syst Rev. 2014 Dec 6;3:142. doi: 10.1186/2046-4053-3-142 (PMC4326322; doi:10.1186/2046-4053-3-142)
Supplement: Supplementary file 10 [file 13643_2014_328_MOESM5_ESM.docx]

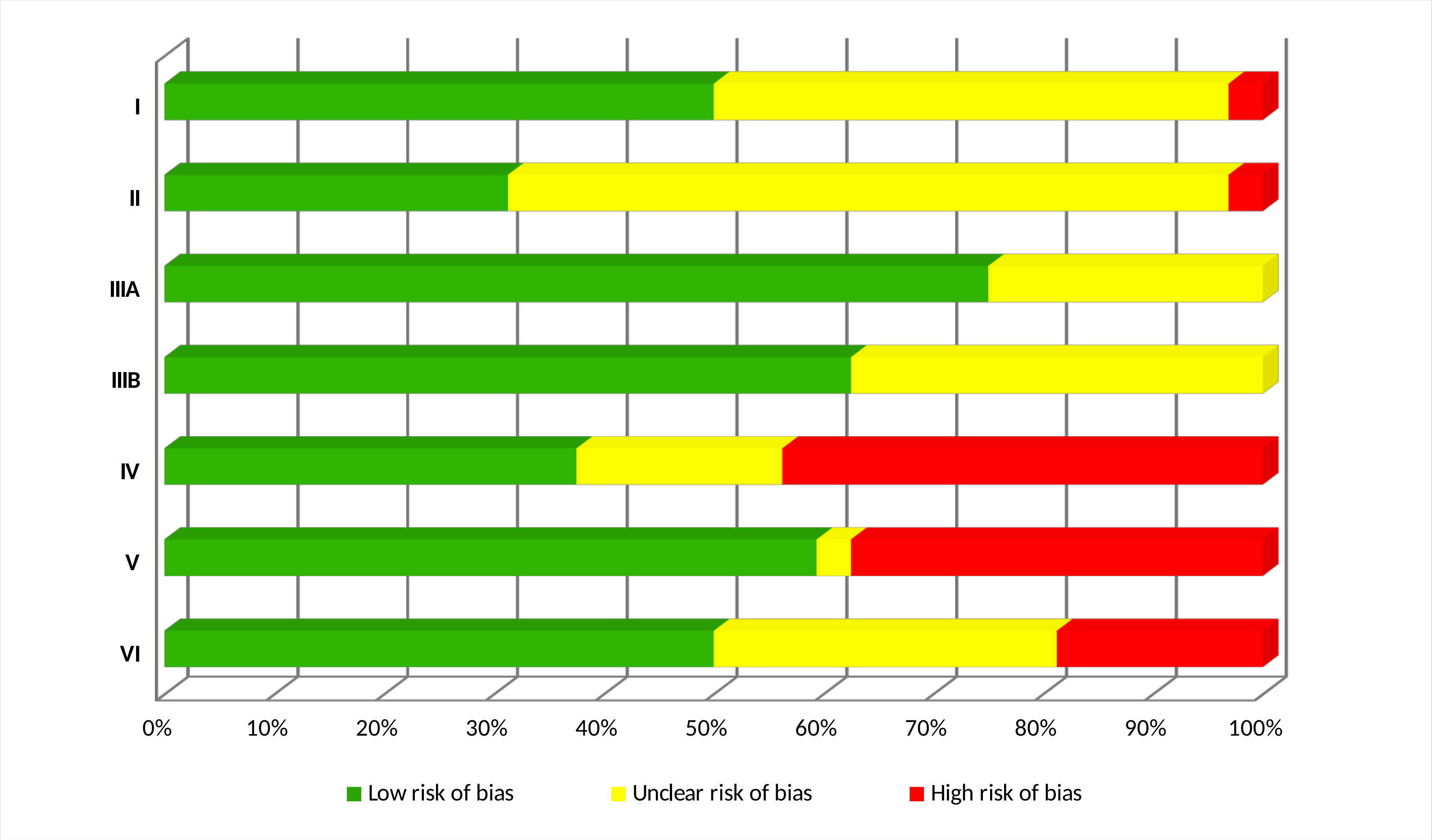
I
II
IIIA
IIIB
IV
V
VI
0%
10%
20%
30%
40%
50%
60%
70%
80%
90%
100%
Low risk of bias
Unclear risk of bias
High risk of bias
